# Online Forums as a Tool for Broader Inclusion of Voices on Health Care Communication Experiences and Serious Illness Care: Mixed Methods Study

**DOI:** 10.2196/48550

**Published:** 2023-12-06

**Authors:** Carine Davila, Stephanie H Chan, Anna Gosline, Zamawa Arenas, Jane Kavanagh, Brian Feltz, Elizabeth McCarthy, Tyrone Pitts, Christine Ritchie

**Affiliations:** 1 Division of Palliative Care and Geriatric Medicine Massachusetts General Hospital Boston, MA United States; 2 Department of Medicine Harvard Medical School Boston, MA United States; 3 Massachusetts Coalition for Serious Illness Care Boston, MA United States; 4 Blue Cross Blue Shield of Massachusetts Boston, MA United States; 5 Flowetik Boston, MA United States; 6 Ariadne Labs Boston, MA United States; 7 3D Research Partners LLC Harvard, MA United States; 8 Elizabeth M McCarthy Consulting Boston, MA United States; 9 The Coalition to Transform Advanced Care Washington, DC United States; 10 Center for Aging in Serious Illness Mongan Institute Boston, MA United States

**Keywords:** serious illness care, serious illness communication, mixed methods research, community-engaged design, equity in research, online forum, health care experiences, internet, illness, marginalized community, efficacy, communication, engagement, quantitative survey, health care

## Abstract

**Background:**

Existing health care research, including serious illness research, often underrepresents individuals from historically marginalized communities. Capturing the nuanced perspectives of individuals around their health care communication experiences is difficult. New research strategies are needed that increase engagement of individuals from diverse backgrounds.

**Objective:**

The aim of this study was to develop a mixed methods approach with qualitative online forums to better understand health communication experiences of individuals, including people from groups historically marginalized such as Black and Latino individuals; older adults; and people with low income, disability, or serious illness.

**Methods:**

We used a multiphase mixed methods, community-informed research approach to design study instruments and engage participants. We engaged a diverse group of collaborators with lived experience of navigating the health care system who provided feedback on instruments, added concepts for testing, and offered guidance on creating a safe experience for participants (phase 1). We conducted a national quantitative survey between April and May 2021 across intrapersonal, interpersonal, and systems-level domains, with particular focus on interpersonal communication between patients and clinicians (phase 2). We conducted two asynchronous, qualitative online forums, a technique used in market research, between June and August 2021, which allowed us to contextualize the learnings and test concepts and messages (phase 3). Using online forums allowed us to probe more deeply into results and hypotheses from the survey to better understand the “whys” and “whats” that surfaced and to test public messages to encourage action around health.

**Results:**

We engaged 46 community partners, including patients and clinicians from a Federally Qualified Health Center, to inform study instrument design. In the quantitative survey, 1854 adults responded, including 50.5% women, 25.2% individuals over 65 years old, and 51.9% individuals with low income. Nearly two-thirds identified as non-Hispanic white (65.7%), 10.4% identified as non-Hispanic Black, and 15.5% identified as Hispanic/Latino. An additional 580 individuals participated in online forums, including 60.7% women, 17.4% individuals over 65 years old, and 49.0% individuals with low income. Among the participants, 70.3% identified as non-Hispanic white, 16.0% as non-Hispanic Black, and 9.5% as Hispanic/Latino. We received rich, diverse input from our online forum participants, and they highlighted satisfaction and increased knowledge with engagement in the forums.

**Conclusions:**

We achieved modest overrepresentation of people who were over 65 years old, identified as non-Hispanic Black, and had low income in our online forums. The size of the online forums (N=580) reflected the voices of 93 Black and 55 Hispanic/Latino participants. Individuals who identify as Hispanic/Latino remained underrepresented, likely because the online forums were offered only in English. Overall, our findings demonstrate the feasibility of using the online forum qualitative approach in a mixed methods study to contextualize, clarify, and expound on quantitative findings when designing public health and clinical communications interventions.

## Introduction

The COVID-19 pandemic raised the US health care community’s acknowledgement of both the historic and current inequities in health care access, treatment, and outcomes. The pandemic therefore highlighted the need for increased engagement of diverse communities to increase the validity of research studies [1-3]. This is true for all of health care, and serious illness research is no exception [3-7]. Serious illness communication (SIC) describes conversations that occur between patients with serious illness and clinicians to understand the patient’s goals, values, preferences, and priorities so that health care can be aligned with those priorities [8]. SIC is a type of shared decision-making and part of the broad set of activities known as advance care planning (ACP). SIC represents an important tool for the creation of therapeutic alliance and has the potential to align goals and clinical decisions. Such conversations are enhanced when clinicians within care systems have the cultural skills, attitudes, behaviors, and interactional styles to promote effective SIC [9,10]. However, there are documented disparities by race, ethnicity, and income in SIC and ACP-related activities, including health care proxy completion rates, conversations with clinicians and family about wishes for care, and the kinds of language that worked best when it comes to encouraging these activities [11-18].

Earlier work from the Massachusetts Coalition for Serious Illness Care (MCSIC) focused on how to best promote SIC and ACP to the public, especially to historically marginalized communities most likely to experience poor health outcomes [19]. Some of the open-ended qualitative research found confusion and misunderstanding about the language used to describe the many activities that are collectively referred to as ACP. It also became clear that many individuals associate the entire field of SIC and ACP with the very end of life and death. No matter how items were phrased, people continuously assumed that the topic was related specifically to do-not-resuscitate orders, “pull the plug” decisions, and what has come to be referred to as “true” end-of-life planning, such as estate or funeral planning [20]. Our goal for this research was to better contextualize people’s beliefs and attitudes about serious illness, SIC, and ACP within the larger canvas of their overall health care experiences. Unlike our prior work, we were not aiming to directly encourage SIC or ACP, but instead to further understand how SIC and ACP might align with the challenges and needs as seen by patients and to establish an approach to generate new insights into how and when ACP and SIC should be introduced and encouraged. Toward this end, we sought new approaches to obtain broader and more nuanced input from historically marginalized communities and ask new questions that solicited a more holistic focus on the health care journey, rather than exclusively on the serious illness journey or end of life journey. Accordingly, our specific question was as follows: How do we engage a wide range of insights on these issues to ensure that the perspectives of a small number of individuals are not extrapolated to reflect entire communities?

Our research had four key aims. The first aim was to understand individuals’ experiences with the health care system that shape care expectations and attitudes toward the system, specifically with regard to medical decision-making, recognition of and support for social determinants of health, and trust in and respect by clinicians and health care systems. Second, we sought to understand the greatest perceived medical, social, and financial needs when it comes to improving serious illness care in the United States. Third, we wanted to understand how individuals perceive sample language that clinicians may use to engage and support individuals under their care and understand what authentically resonates with them. Finally, we wanted to obtain input on how best to contextualize and frame different public messages to encourage action around health, including SIC and ACP. We sought to explicitly understand these perspectives of individuals from historically marginalized communities, who are often underrepresented in research.

Here, we describe our overarching approach to optimize representation in the research, demonstrate the breadth of engagement in our quantitative survey and qualitative online forums, and highlight participants’ satisfaction with the online forum engagement tool.

## Methods

### Overview

**Figure 1:**
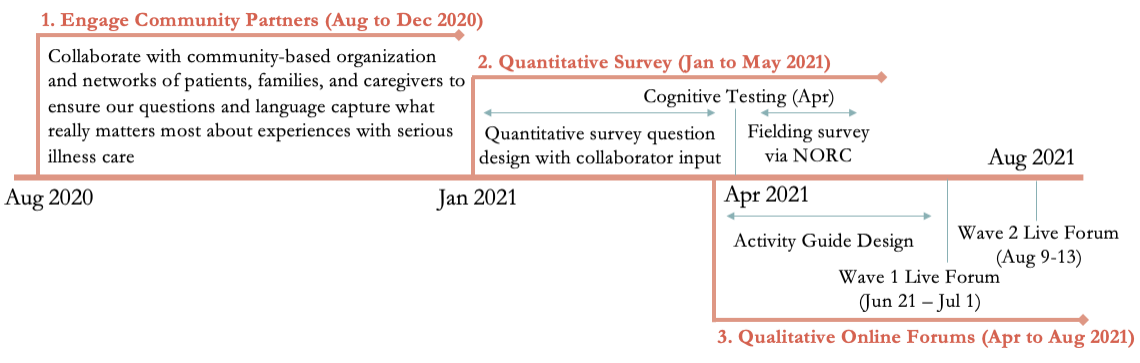
Research timeline spanning three phases in the mixed methods study, August 2020-August 2021. NORC: National Opinion Research Center.

We designed a mixed methods community-informed research study. The study took place in three phases from August 2020 to August 2021 (see Figure 1). Guided by our aims, we sought to understand: What are people’s lived experiences in health care settings? What are the challenges faced by people with serious illness and caregivers? How does this impact what we should prioritize saying, doing, and asking people to do?

### Ethical Approval

This study received approval through the Harvard Longwood Campus Institutional Review Board (phase 2, IRB21-0398) and Massachusetts General Brigham’s Institutional Review Board (phase 3, 2021P003549).

### Phase 1: Engage Community Partners in Study Design

Our goal was to gather input from community partners to ensure that our research objectives, approach, and framing were aligned with the needs of people from historically marginalized communities. We wanted to understand what matters to people around serious illness care, trying to discard our biases and assumptions. We engaged people through various channels, including focus groups with clinicians at a Federally Qualified Health Center (FQHC), in-depth telephone interviews with Black and Latino low-income older adults who participate in a Program for All-Inclusive Care for the Elderly at the FQHC, and an online survey with input from leading serious illness care organizations across the country. All participants were compensated for their time and engagement. We asked FQHC participants what situations and circumstances impact the type of health care Black and Latino patients receive, what they are most concerned about regarding their health and health care, and what they thought needed to change to improve people’s health care experiences. We asked clinicians and serious illness care leaders what and how to ask about people’s health care experiences, including SIC and ACP, and what needs to change to improve these health care experiences. These insights informed the development of study instruments for both our quantitative survey (phase 2) and qualitative online forum guide (phase 3). By the end of phase 1, we had a small diverse group of collaborators with lived experience of the challenges people face navigating the health care system, who provided more detailed and focused feedback on each instrument, added new concepts for testing, and offered guidance on creating a safe and caring experience for our participants. This group included additional MCSIC members and other national leaders in serious illness care, representing a diverse group of voices as listed in the Acknowledgments.

### Phase 2: Quantitative Survey

#### Participant Recruitment

Survey respondents were included and invited to participate via the National Opinion Research Center (NORC) at the University of Chicago through their national AmeriSpeak panel. The AmeriSpeak panel is a probability-based household panel that uses a multistaged probability-based sampling method through which NORC achieved an estimated sample frame coverage of 97% of the residential United States, including a supplemental list of rural households not recorded on the US Postal Service Computerized Delivery Sequence file but identified through NORC in-person fieldwork. Households are sampled with a known, nonzero probability of selection from the NORC National Frame and recruited through a rigorous process that uses mail, telephone, and in-person recruitment by field interviewers to ensure that even hard-to-reach populations are represented in the panel [21-23]. Enrollment targets were set at a minimum of 100 respondents for specific groups: people with low income (less than US $50,000/year), Black respondents with low income, Latino respondents with low income, age greater than 65 years, people with disability (self-identified and/or answering “yes” to any of six questions about function from the American Community Survey, hereafter described as ACS-6 [24,25]), and people with serious illness (as per the “Identifying People With Serious Illness” subsection below). No specific exclusion criteria were designated. In addition to the national sample described here, a nonprobability-based sample of Massachusetts-based residents was administered through Lucid for separate state-specific analyses [26].

#### Quantitative Survey Development

**Figure 2:**
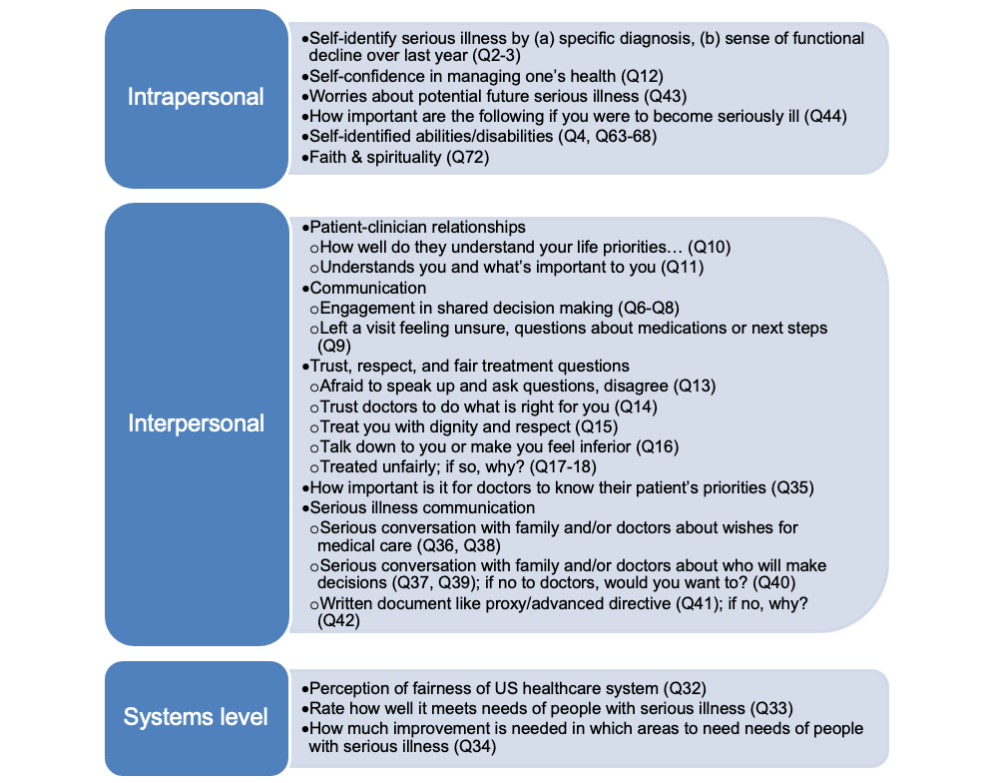
Quantitative survey domains, based on the social ecological model; phase 2 of the mixed methods study.

The design of the quantitative survey was informed by extensive review of the literature, engagement with community partners in phase 1, and the social ecological model [27]. The survey instrument covered topics across intrapersonal, interpersonal, and systems-level domains (Figure 2). We particularly focused on the interpersonal domain between patients and clinicians, covering topics such as whether patients feel engaged in shared decision-making, trust clinicians to do what is right, feel treated with dignity and respect, feel afraid to speak up and ask questions, or feel talked down to or made to feel inferior. Complementing these domains, we iteratively engaged our ongoing collaborators on question topics and language, including how we identified people with serious illness (see below).

NORC conducted eight cognitive interviews from AmeriSpeak panelists by video to qualitatively understand how the survey questions were interpreted and make recommendations on alternative word choices. These interviews yielded meaningful recommendations to simplify language, add in clarifiers, provide additional answer choices that respondents thought were missing, and add appropriate prompts to facilitate the flow of the survey. The full quantitative survey instrument is included in Multimedia Appendix 1.

#### Identifying People With Serious Illness

We iteratively engaged with our research and community partners and determined a need to distinguish people with serious illness from people with chronic disability. We identified people with serious illness based on whether participants self-reported “yes” to two questions: (1) have you ever been diagnosed with any of the following (diabetes; asthma, lung disease, emphysema, or chronic obstructive pulmonary disease; heart disease or had a stroke; cancer; Alzheimer disease, dementia, or memory loss; depression, anxiety, or other serious mental health problems; or chronic kidney disease or kidney failure)?, and (2) over the last 12 months, would you say that you have been feeling sicker and that it’s been getting harder to do your normal levels of work and activity?

#### Survey Fielding

NORC AmeriSpeak panelists were invited to participate in the survey between April 20, 2021, and May 17, 2021. The survey was offered in English and Spanish and via both the web and telephone to adults aged 18 years and older. Web-mode panelists were sent up to five email reminders to encourage participation and phone-mode panelists were called throughout the field period. Panelists were offered the cash equivalent of US $3 for completing the survey.

### Phase 3: Qualitative Online Forums

#### Description

**Figure 3:**
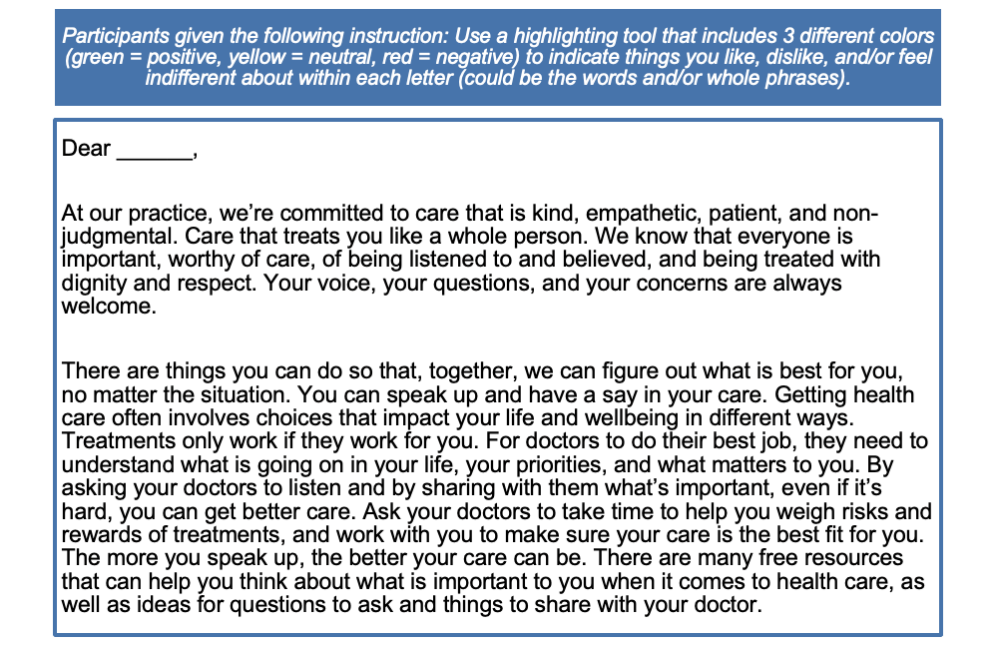
Online forum sample message to encourage action; phase 3 of the mixed methods study.

To supplement a traditional quantitative survey, we elected to use a series of asynchronous, qualitative online forums, a technique commonly used in market research to gain deeper understanding of why individuals think, believe, and feel how they do. We engaged participants who were part of online forums to delve deeper into understanding people’s health care experiences, testing sample public messages with participants, and framing different public messages that encourage action around SIC and ACP in the context of people’s lived experiences (see Figure 3). Online forums allow engagement of a wide variety of individuals with sample sizes large enough to ensure varied perspectives even within groups that are historically underrepresented and marginalized.

#### Participant Recruitment

Participants were recruited by Full Circle Research Co, an independent online participant sample provider [28]. Individuals participating with the sample provider completed a screening questionnaire including demographics to determine eligibility. Inclusion criteria included availability to participate during scheduled online forum dates, aged 18 years and older, and having convenient access to a computer or smartphone with a high-speed or broadband internet connection. Exclusion criteria included lack of appropriate technology (such as convenient access to a device and the internet and an up-to-date internet browser such as Microsoft Internet Explorer Version 7.0 or higher, Mozilla Firefox Version 3.0 or higher, Safari Version 2.0.4 or higher, or any version of Google Chrome), inability to participate in the online forum in English (requiring ability to speak English and ability to type free-text responses), and less than high school education.

Participants were recruited to represent a mix of individuals across age, gender/gender identity, geography/state, education, marital status, employment status, race/ethnicity, income, and self-described health status. Individuals from the following groups were intentionally oversampled: people with low income (less than US $50,000/year), Black respondents with low income, Latino respondents with low income, age greater than 65 years, people with disability, people living with disability (as per phase 2 and ACS-6 [24,25]), people with serious illness (as per the identification criteria outlined in phase 2), and caregiver (defined as helping someone close to them who has a lot of medical or health needs or conditions). We sought to oversample these groups to ensure diversity of perspectives informing our findings.

#### Online Forum Activity Guide Design

Structured activity guides (see Multimedia Appendix 2 and Multimedia Appendix 3) were designed to engage participants in the online forum. Activity guide design was deeply informed by input from community partner engagement in phase 1 and coincided with receiving preliminary results from the phase 2 quantitative survey. Therefore, some activity prompts were created to probe deeper into specific questions, results, or hypotheses generated from the quantitative survey, allowing us to better understand the “whys” in addition to the “whats” that surfaced in the quantitative survey. Each online forum began with “getting to know you” activities to quickly establish trust and inspire participants to see their responses as more than answering research questions. Most activities were open-ended conversation prompts. To encourage continued engagement and reduce response fatigue, other activity formats were used, including answering multiple-choice questions; asking participants to tell us why they chose certain responses; reacting to definitions of quality care; rating the perceived impact of specific language clinicians may use to engage individuals in health care decision-making; and reading through sample public messages on actions one can take to address health and well-being with a highlighter tool to indicate words and phrases that stood out as being positive, negative, or neutral.

#### Online Forum Fielding

**Figure 4:**
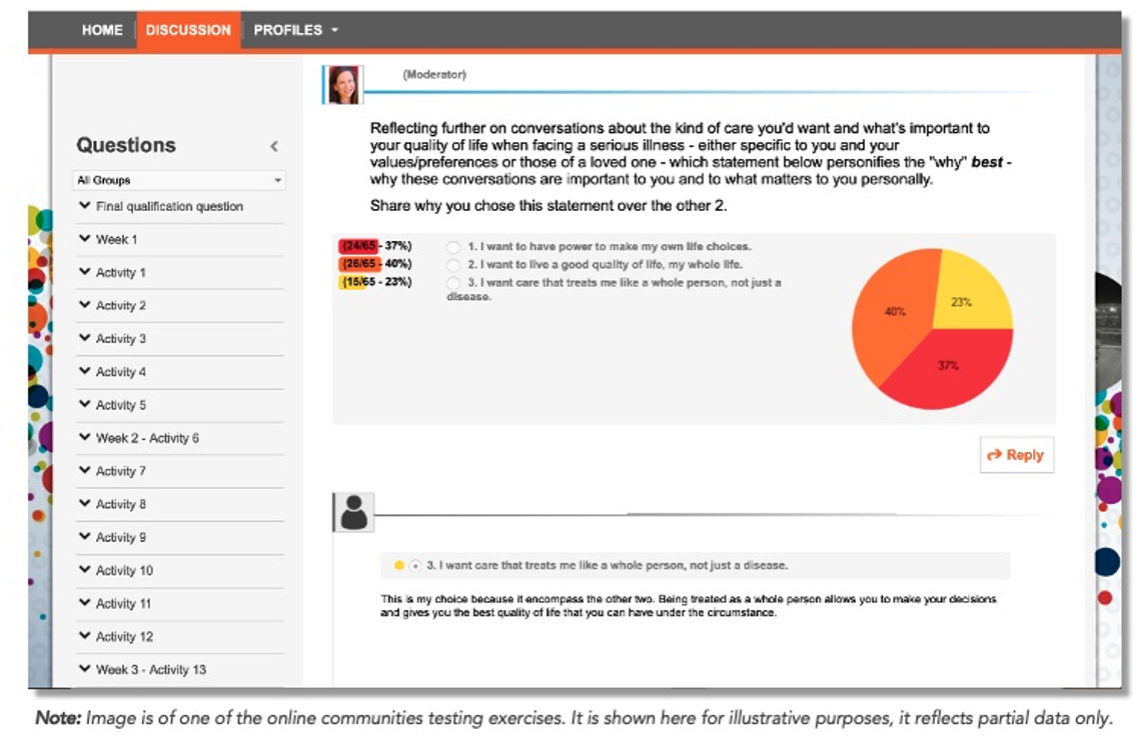
Sample activity on the itracksBoard online forum platform; phase 3 of the mixed methods study.

Qualified individuals were invited to participate in the online forums via itracksBoard, an online qualitative research platform [29]. Each week’s activities were designed to take around 2 hours to complete over the course of a 5-day week, with new activities available twice per day for a total of 7 or 8 activities per week (see Figure 4 for a sample activity). Participants responded to prompts asynchronously. A moderator (EM) published these activity prompts and then was present in the forum by asking probing, follow-up questions to the participants to encourage more conversation.

The online forums were administered in two waves. Wave 1 was conducted for 2 weeks from June 21, 2021, to July 1, 2021, targeting 250 participants in two communities (targeting no more than 125-150 individuals in each community). The primary objective of the first wave was to explore consumers’ health care experiences, both good and bad; the drivers that contributed to these experiences; and reactions to sample messaging clinicians could use to engage patients in their care. Wave 2 was 1-week long from August 9, 2021, to August 13, 2021, targeting 250 participants with three communities (to allow testing with three different messaging frames). The second wave explored how well the participants feel known by their clinicians, their concerns about future care if they became ill, and their reactions to messaging encouraging them to act as one of three options: as a public health campaign, a simple doctor’s letter, and a doctor’s office letter with additional framing language. This sample messaging was developed and informed by the quantitative survey findings. The forums were purposefully smaller in the second wave to test different messages with distinct populations. Participants who completed all activities in wave 1 received a US $125 gift card and those that completed the activities in wave 2 received a US $100 gift card.

### Analysis

#### Phase 2: Quantitative Survey

To account for differences in nonresponse, NORC applied statistical weighting to adjust to Current Population Survey totals associated with age, gender, education, race/Hispanic ethnicity, housing tenure, telephone status, and census division. Additional sampling weights were applied to account for the interactions of age and gender, age and race/ethnicity, and race/ethnicity and gender. The weighted data, which reflects the US population of adults aged over 18 years, were used in subsequent analyses (not shown here). Descriptive statistics were calculated using SPSS version 29 (IBM Corp, Armonk, NY, USA).

#### Phase 3: Qualitative Online Forums

Two authors (EM and ZA) conducted qualitative content analysis of online forum data [30]. We conducted the qualitative analysis in a hybrid inductive-deductive manner based on a combination of the domains from the activity guides and the information that emerged from the online responses. This involved separately combing through daily participant responses verbatim, which comprised responses to research questions intentionally fielded from the activity guide and the organic conversation participants engaged in, all within the “walls” of the online forum, and identifying observations deemed consistent, insightful, and worthy of more exploration. We then compared high-level observations and grouped these into key themes that addressed the overall research questions. We created summaries of participants’ responses by activity, identifying important points, and highlighting key themes for review.

## Results

### Phase 1: Engage Community Partners

We engaged with 46 community partners through various channels. We conducted two focus groups with seven clinicians and care navigators from an FQHC community health center, six in-depth telephone interviews with Black and Latino low-income older adults who receive care at an FQHC, and 12 respondents to an online survey with representatives from leading serious illness care organizations across the country. We also received additional input from 21 MCSIC members and other national leaders in serious illness care.

Our conversations from care providers and people with lived serious illness experience yielded many common experiences and concerns with health care systems that impact how historically marginalized groups experience serious illness care (see Textbox 1). People shared challenges in navigating the health care system; the unaffordability of care; experiences with bias and discrimination; the geographic inaccessibility of care; and the limited ability clinicians have to address more pressing social challenges impacting their health, including housing, food insecurity, immigration status, and loss of income. These findings led us to focus our study instruments on people’s prior health care experiences and how their current care fits into other life priorities.

**Key themes from community partners (N=46), comprising US adults engaged from February to July 2021, in phase 1 of the mixed methods study.**

- Difficulty navigating the health care system, such as managing referrals to multiple specialists, accessing home care, and getting prescriptions refilled.
- Unaffordability of care (including the cost of health insurance and medications) as having a real impact on well-being.
- Experiencing bias and discrimination based on a variety of factors, including race, immigration status, age, disability, insurance coverage, and more.
- Lack of high-quality care geographically close by and transportation challenges getting to appointments far from where they live.
- Clinicians are not trained to help beyond immediate medical needs and those other needs are often much more pressing, such as housing, food insecurity, immigration status, and loss of income.

### Phase 2: Quantitative Survey

The survey was fielded to 6126 households with a survey completion rate of 30.3%. The margin of error was ±3.08 percentage points and the median duration to complete the survey was 12 minutes. The population consisted of 1854 adults (955 women, 51.5%; mean age 48.4, SD 17.5 years). Table 1 shows the demographics of the survey respondents, with both unweighted and weighted values. Of 1854 surveys, 94.5% occurred online (not by phone) and 2% occurred in Spanish. The respondents were 65.7% white (non-Hispanic), 10.4% Black (non-Hispanic), and 15.5% Hispanic. Approximately 1 in 5 (19.8%) respondents were categorized as having a serious illness and 18.9% self-identified as having a disability.

**Table 1:** Quantitative survey population characteristics (N=1854), comprising a nationally representative sample of US adults fielded from April to May 2021, in phase 2 of the mixed methods study.

Characteristic			Unweighted value, n (%)		Weighted value, %
Gender: woman			955 (51.5)		51.9
**Age (years)**					
	18-24	70 (3.8)		7.0	
	25-34	415 (22.4)		22.5	
	35-44	292 (15.7)		17.3	
	45-54	255 (13.8)		12.8	
	55-64	354 (19.1)		18.7	
	65-74	325 (17.5)		14.9	
	75+	143 (7.7)		6.8	
**Race/ethnicity**					
	White, non-Hispanic	1218 (65.7)		61.2	
	Black, non-Hispanic	193 (10.4)		12.0	
	Asian, non-Hispanic	50 (2.7)		6.5	
	>2 races or other, non-Hispanic	105 (5.7)		3.7	
	Hispanic	288 (15.5)		16.7	
**Annual household income (US $)**					
	<30,000	437 (23.6)		17.8	
	30,000 to <60,000	526 (28.4)		25.1	
	60,000 to <100,000	464 (25.0)		30.1	
	≥100,000	427 (23.0)		26.9	
**Health insurance status**					
	Employer or spouse’s employer	807 (43.5)		44.9	
	Medicare	412 (22.2)		19.9	
	Other	447 (24.1)		23.7	
	No insurance	188 (10.1)		11.5	
Current employment status: working			1079 (58.2)		59.1
**Education**					
	Less than high school	58 (3.1)		9.8	
	High school graduate or equivalent	317 (17.1)		27.8	
	Vocational/technical school/some college/associates degree	794 (42.8)		27.6	
	Bachelor’s degree	386 (20.8)		19.6	
	Postgraduate study/professional degree	299 (16.1)		15.2	
People with disability			351 (18.9)		15.6
**Region**					
	Northeast	256 (13.8)		17.3	
	Midwest	491 (26.5)		20.7	
	South	628 (33.9)		38.0	
	West	479 (25.8)		23.9	

### Phase 3: Online Forums

A total of 5191 individuals received the screening questionnaire, with 1512 qualifying individuals invited to participate. Of the 1512 individuals invited to participate in the forums, 644 (42.6%) joined and 90.0% (n=580) completed every activity and comprised the participant pool. Online forum participants’ demographics are outlined in Table 2. Approximately 60.7% of participants identified as women, compared to 50.5% of the US population [31]. An estimated 70.3% of participants were white (non-Hispanic), 16.0% Black (non-Hispanic), 9.5% Hispanic, and 1.9% Asian, compared to census estimates of 59.3%, 13.6%, 18.9%, and 6.1%, respectively [31]. Approximately 15.9% of the participants were categorized as having a serious illness, 15.2% self-identified as having a disability, and 18.4% self-identified as a caregiver.

**Table 2:** Qualitative online forum participant characteristics (N=580), comprising a national sample of US adults fielded from June to August 2021, in phase 3 of the mixed methods study.

Characteristics			Participants, n (%)^a^
Gender: woman			352 (60.7)
**Age (years)**			
	18-24	17 (2.9)	
	25-34	83 (14.3)	
	35-44	85 (14.7)	
	45-54	107 (18.4)	
	55-64	162 (27.9)	
	65-75	90 (15.5)	
	75+	11 (1.9)	
	Unknown	25 (4.3)	
**Race/ethnicity**			
	White, non-Hispanic	408 (70.3)	
	Black, non-Hispanic	93 (16.0)	
	Asian, non-Hispanic	11 (1.9)	
	>2 races or other, non-Hispanic	13 (2.2)	
	Hispanic	55 (9.5)	
**Annual household income (US $)**			
	<25,000	111 (19.1)	
	25,000 to <50,000	173 (29.8)	
	50,000 to <100,000	168 (29.0)	
	≥100,000	103 (17.8)	
	Unknown	25 (4.3)	
People with disability			88 (15.2)
**Region**			
	Northeast	187 (32.2)	
	Midwest	94 (16.2)	
	South	217 (37.4)	
	West	82 (14.1)	

^a^Percentages may not all add to 100% due to rounding.

Furthermore, participants highlighted that they enjoyed the opportunity to engage in manner through online forums, that they learned from one another and their experiences, and that some even felt motivated to take action in their own health journey (see Textbox 2).

**Reflections from online forum participants, comprising a national sample of US adults fielded from June to August 2021, in phase 3 of the mixed methods study.**

*Attitudes*

- “I enjoyed participating in this study. Thank you for the opportunity to voice my opinions.” [middle-aged multiracial woman with serious illness and disability]
- “Thank you for this fun group. I learned a lot from the other members and was glad I was able to contribute my thoughts. I really felt heard.” [young Latina woman with comorbid illness]

*Knowledge*

- “This was a great group to be in. I appreciated everyone’s thoughts/comments. Some of them were some real eye openers. Thank you for allowing me to be a part of this group. I appreciated it.” [older white woman with disability]
- “It has been not only a pleasure but also a learning experience for me as well. It made me think of health issues that could and will arise as time goes by and it’s good to have plans and invest time in surrounding yourself and family with a health care environment that will be proactive about your care and well being. Again my thanks to you Beth and the Coalition.” [older Black man]

*Behaviors (engagement/activation)*

- “It has inspired me to not be afraid to talk up and give my opinions regarding whatever situation but especially health care. Only because I used to be so nervous walking into a doctor’s office. Now, I’ve learned to put my fears aside and ask questions and express myself because that is the only way my doctor is able to know me better and prescribe my treatments.” [older woman with serious illness]
- “For a while I have been procrastinating about designating a proxy to speak for me regarding health care decisions…and getting a will. Why? Because like most human beings I want to concentrate on life in the present instead of worrying too much about sickness and death in the future. It is a mechanism of defense we human beings have. The discussions here addressing these topics made me realize that I have to take action now, grab the bull by the horns as they say. These decisions and actions are not easy but necessary because even if we are healthy and alive today we never know what can happen tomorrow. I have to take action. For starters, I have to think carefully about who will be my health care proxy and I have to work on getting a will.” [younger man with comorbid illness]

## Discussion

Understanding people’s health care communication experiences in the past is important as these experiences will influence their willingness to seek health care services [32] and in turn engage in future serious illness conversations. SIC strategies improve quality of life, enhance communication quality, reduce psychological distress, and promote positive patient and clinician experiences [33-38]. This community-informed mixed methods research study utilized online forums to better understand how SIC and ACP fit in the context of people’s prior experiences and life priorities. This study presents a unique approach with online engagement that is feasible and offers benefits over other traditional research approaches. We engaged 46 individual community partners, including patients, clinicians, and serious illness national experts, in our first phase of research, obtaining rich and compelling feedback that we incorporated in the design of study instruments. In our quantitative second phase, we used an intentionally recruited census-representative cohort of US adults through the NORC AmeriSpeak panel to reach over 1850 adults, which is in line with the recent large Kaiser Family Foundation National Serious Illness Care Survey with 2000 respondents [15]. Our survey population included nearly 20% respondents who have a serious illness, which is a greater proportion than included in the Kaiser Family Foundation’s survey, and 19% who have a disability, which is below the 25.7% estimated prevalence of US adults who live with at least one disability [39]. Our survey population also included 1386 (74.7%) younger and middle-aged adults (less than 65 years), who are often not captured relative to health care lived experience, beliefs, and concerns related to serious illness care. In our qualitative phase, we used asynchronous online forums as a method of engaging 580 US adults, a much larger number of people than traditional qualitative method styles can obtain, to ensure that the voices of a few are not used to represent entire historically marginalized populations. Recruitment for the online forums focused on engaging individuals from historically marginalized communities that are often underincluded in research efforts. In recruitment of diverse populations, it is important to assure compensation [40]. In all phases of our work, including online forums, we provided reimbursement for participation. With intentional efforts to oversample people from historically marginalized communities, we achieved overrepresentation of individuals who are over 65 years old, identify as non-Hispanic Black, and who have an annual income below the median US household income in the online forum population. We remained underrepresented in terms of individuals who identify as Hispanic/Latino and non-Hispanic Asian in our online forum population.

Our study achieved mixed results in terms of diverse racial/ethnic participant engagement in the quantitative and qualitative phases. In the quantitative phase, where we relied on the prerecruited panel of potential participants, we achieved lower than census levels of representation from non-Hispanic Black (10.4% vs 13.6% in the census), Hispanic/Latino (15.5% vs 18.9% in the census), and non-Hispanic Asian (2.7% vs 6.1% in the census) individuals [31]. These findings reflect that even when utilizing a census-representative survey sampling population, additional efforts may be needed to achieve census-level representation of historically marginalized racial/ethnic communities. In the qualitative phase, where we conducted intentional oversampling of Black and Latino individuals, we achieved higher than census levels of representation of non-Hispanic Black individuals (16.0% vs 13.6% in the census) and lower than census levels of representation of Hispanic/Latino (9.5% vs 18.9% in the census) and Asian (1.9% vs 6.1% in the census) individuals [31]. Importantly, the large size of the online forum population (N=580) reflected 93 Black and 55 Hispanic/Latino participants who were included in the study, ensuring that the perspectives of only a small handful of individuals are not extrapolated to reflect an entire historically marginalized community. This online forum recruitment highlights that intentional oversampling can achieve overrepresentation of some populations, including non-Hispanic Black individuals, but additional effort to offer participation in Spanish may improve engagement of Hispanic/Latino individuals, as two out of three Latino individuals report Spanish as their language preference [41], and Asian individuals, who have also been historically underincluded and often grouped even though they reflect a wide variation of languages, cultures, and backgrounds [42].

Prior studies have qualitatively examined patients’ health care experience and others have utilized publicly available online forum comments for qualitative analysis [43,44]. There has been some use of online forums to qualitatively collect information from patients [43-47], although here we describe the benefit of using online forums that allowed for tailored recruitment and greater representation from groups that are historically marginalized. This mixed methods approach, especially with such a flexible design for how to engage participants in online forums, allowed for rich, people-centered results in a scalable format. The recruitment approach engaged individuals from specific subgroups whose voices are not always elevated in research, including individuals from some historically marginalized communities (eg, 93 Black individuals and 55 Hispanic/Latino individuals) although notably not all (eg, only 11 Asian individuals participated). This helped to ensure that we heard varied perspectives within groups as well as between groups. The size and interactive nature of the forums allowed participants to surface themes that we did not know to ask about, such as weight bias, and probe concurrently through the moderator as they arose. We were also able to explore survey questions we needed further insights on to interpret the quantitative survey responses.

Finally, participants shared that they were engaged and satisfied with the online forum engagement process, some even sharing that they felt inspired to do new or different things (eg, finding a new doctor or speaking up more at a visit) given the content they were exposed to during the forums through the structured exercises as well as from interacting with each other. This feedback informally suggests that participation in this study increased their patient activation, although formal assessment of the Patient Activation Measure was not conducted. Prior research by Hibbard and Greene [48] outlined that higher patient activation is linked with improved health outcomes. Furthermore, the same research group highlighted that when patient activation increases, it is frequently associated with improved health outcomes and lower costs [49]. Participants’ positive reflections on their online forum experience suggest that this is a feasible way to engage a wide range of perspectives in qualitative research.

There are also study limitations that should be mentioned. The quantitative survey was fielded in English and Spanish, as well as electronically online and by telephone, although respondents were limited to those participating in NORC’s AmeriSpeak panel, which may inherently represent a population typically more willing to engage in survey research. The survey completion rate was 30%, which is in line with probability-based survey panel completion rates using the NORC AmeriSpeak panel [50]. Statistical weighting was used to overcome sampling bias by adjusting to the Current Population Survey and for interactions. In our qualitative online forums, participation was likely limited by the forums being conducted only in English, online only, and requiring typing for many of the activities. This likely contributed to underrepresentation of Hispanic/Latino and non-Hispanic Asian participants and possibly contributed to less engagement from people with disability, who may experience challenges with eyesight and typing ability required to engage in this forum. Future studies could consider offering a parallel forum in Spanish and ensuring browser/forum compatibility with text-to-voice and voice-to-text software for individuals with eyesight or typing challenges.

There are important opportunities for future research. In addition to highlighting the recruitment of individuals from historically marginalized communities in this mixed methods study, further research highlighting the differences in people’s prior health care experiences and engagement in SIC and ACP across individual characteristics and identities (eg, race/ethnicity, income, serious illness, and disability) is important. Future qualitative research could consider using a parallel online forum offered in Spanish to engage perspectives from Hispanic/Latino individuals who prefer Spanish. It would also be helpful to better understand the barriers and facilitators for people from historically marginalized communities to engage in serious illness conversations, building on prior work exploring barriers and facilitators to SIC among Black individuals and increasing understanding for Hispanic/Latino and Asian individuals [51].

Here, we outline a community-informed mixed methods approach to understanding people’s prior health care experiences and testing the framing of public messages to encourage engagement with clinicians and action around health, in service of finding new strategies to improve serious illness care. Our approach of engaging the community throughout the study design and execution focused our study, enriched our study instruments, and yielded important findings. This study can serve as a feasible model of choosing methodologies that allow for engagement with target communities and in-depth exploration of research aims, which in our case is to better understand how to improve serious illness care and communication.

## Appendix

Multimedia Appendix 1 Phase 2: quantitative survey instrument, March 2021.

Multimedia Appendix 2 Phase 3: online forum activity guide, June 2021.

Multimedia Appendix 3 Phase 3: online forum activity guide, August 2021.

## Abbreviations

ACP: advance care planning
ACS-6: American Community Survey
FQHC: Federally Qualified Health Center
MCSIC: Massachusetts Coalition for Serious Illness Care
NORC: National Opinion Research Center
SIC: serious illness communication

## Data Availability

The data sets generated during and/or analyzed during this study are not publicly available due to terms of consent for study participation but are available from the corresponding author on reasonable request.

## References

[1] Armstrong K, Ritchie C (2022). Research participation in marginalized communities - overcoming barriers. N Engl J Med.

[2] George S, Duran N, Norris K (2014). A systematic review of barriers and facilitators to minority research participation among African Americans, Latinos, Asian Americans, and Pacific Islanders. Am J Public Health.

[3] Barrett NJ, Hasan M, Bethea K, Johnson KS (2020). The fierce urgency of now: addressing racial and ethnic disparities in serious illness care. N C Med J.

[4] Mack JW, Paulk ME, Viswanath K, Prigerson HG (2010). Racial disparities in the outcomes of communication on medical care received near death. Arch Intern Med.

[5] Johnson KS (2013). Racial and ethnic disparities in palliative care. J Palliat Med.

[6] Frydman JL, Gelfman LP, Morillo J, Allen OS, Bickell NA, Kwon D, Pollak KI, Smith CB (2022). Racial/ethnic disparities in serious illness communication for patients with cancer. J Clin Oncol.

[7] Rhodes RL, Barrett NJ, Ejem DB, Sloan DH, Bullock K, Bethea K, Durant RW, Anderson GT, Hasan M, Travitz G, Thompson A, Johnson KS (2022). A review of race and ethnicity in hospice and palliative medicine research: representation matters. J Pain Symptom Manage.

[8] Sanders JJ, Paladino J, Reaves E, Luetke-Stahlman H, Anhang Price R, Lorenz K, Hanson LC, Curtis JR, Meier DE, Fromme EK, Block SD (2020). Quality measurement of serious illness communication: recommendations for health systems based on findings from a symposium of national experts. J Palliat Med.

[9] Shim JK (2010). Cultural health capital: a theoretical approach to understanding health care interactions and the dynamics of unequal treatment. J Health Soc Behav.

[10] Epstein RM, Street Jr RL (2007). Patient-centered communication in cancer care: promoting healingreducing suffering. Patient-centered communication in cancer care. National Cancer Institute.

[11] Clark MA, Person SD, Gosline A, Gawande AA, Block SD (2018). Racial and ethnic differences in advance care planning: results of a statewide population-based survey. J Palliat Med.

[12] Huang IA, Neuhaus JM, Chiong W (2016). Racial and ethnic differences in advance directive possession: role of demographic factors, religious affiliation, and personal health values in a national survey of older adults. J Palliat Med.

[13] Smith AK, McCarthy EP, Paulk E, Balboni TA, Maciejewski PK, Block SD, Prigerson HG (2008). Racial and ethnic differences in advance care planning among patients with cancer: impact of terminal illness acknowledgment, religiousness, and treatment preferences. J Clin Oncol.

[14] Harrison KL, Adrion ER, Ritchie CS, Sudore RL, Smith AK (2016). Low completion and disparities in advance care planning activities among older Medicare beneficiaries. JAMA Intern Med.

[15] DiJulio B, Hamel L, Wu B, Brodie M (2017). Serious illness in late life: the public's views and experiences. Kaiser Family Foundation.

[16] Nouri S, Lyles CR, Rubinsky AD, Patel K, Desai R, Fields J, DeRouen MC, Volow A, Bibbins-Domingo K, Sudore RL (2020). Evaluation of neighborhood socioeconomic characteristics and advance care planning among older adults. JAMA Netw Open.

[17] Nouri SS, Barnes DE, Volow AM, McMahan RD, Kushel M, Jin C, Boscardin J, Sudore RL (2019). Health literacy matters more than experience for advance care planning knowledge among older adults. J Am Geriatr Soc.

[18] (2018). Massachusetts Survey on Advance Care Planning and Serious Illness Care, Spring 2018 Survey of Massachusetts Residents. Massachusetts Coalition for Serious Illness Care.

[19] (2019). Advancing the language of advance care planning: a messaging research project. Massachusetts Coalition for Serious Illness Care.

[20] 2017 consumer research: deep dive on conversations. Massachusetts Coalition for Serious Illness Care.

[21] AmeriSpeak.

[22] NORC at the University of Chicago.

[23] Technical overview of the AmeriSpeak® panel NORC's probability-based household panel. AmeriSpeak and NORC and the University of Chicago.

[24] (2011). HHS implementation guidance on data collection standards for race, ethnicity, sex, primary language, and disability status. Assistant Secretary for Planning and Evaluation (ASPE).

[25] (2020). Population surveys that include the Standard Disability Questions. Centers for Disease Control and Prevention.

[26] Lucid, a Cint Group company.

[27] Crabtree BF, Miller WL (2012). Doing qualitative research.

[28] (2021). Full Circle Research.

[29] itracks: Insight for growth.

[30] Neergaard MA, Olesen F, Andersen RS, Sondergaard J (2009). Qualitative description - the poor cousin of health research?. BMC Med Res Methodol.

[31] QuickFacts United States. US Census Bureau.

[32] Butler SM, Sheriff N (2021). How poor communication exacerbates health inequities – and what to do about it. Brookings Institution.

[33] Curtis JR, Downey L, Back AL, Nielsen EL, Paul S, Lahdya AZ, Treece PD, Armstrong P, Peck R, Engelberg RA (2018). Effect of a patient and clinician communication-priming intervention on patient-reported goals-of-care discussions between patients with serious illness and clinicians: a randomized clinical trial. JAMA Intern Med.

[34] Bernacki R, Paladino J, Neville BA, Hutchings M, Kavanagh J, Geerse OP, Lakin J, Sanders JJ, Miller K, Lipsitz S, Gawande AA, Block SD (2019). Effect of the serious illness care program in outpatient oncology: a cluster randomized clinical trial. JAMA Intern Med.

[35] Kumar P, Wixon-Genack J, Kavanagh J, Sanders JJ, Paladino J, O’Connor NR (2020). Serious illness conversations with outpatient oncology clinicians: understanding the patient experience. JCO Oncol Pract.

[36] Paladino J, Koritsanszky L, Nisotel L, Neville BA, Miller K, Sanders J, Benjamin E, Fromme E, Block S, Bernacki R (2020). Patient and clinician experience of a serious illness conversation guide in oncology: a descriptive analysis. Cancer Med.

[37] You J, Singh J, Simon J, Ma IW, Paladino J, Swinton M, Kobewka D, Munene P, Jayaraman D, Dunne F, Lagrotteria A, Bernacki R (2022). A quality improvement initiative to implement the Serious Illness Care Program on hospital medical wards. Can J Gen Int Med.

[38] Wright AA, Zhang B, Ray A, Mack JW, Trice E, Balboni T, Mitchell SL, Jackson VA, Block SD, Maciejewski PK, Prigerson HG (2008). Associations between end-of-life discussions, patient mental health, medical care near death, and caregiver bereavement adjustment. JAMA.

[39] (2019). Prevalence of disability and disability types by urban-rural county classification - United States, 2016. Centers for Disease Control and Prevention.

[40] Bierer BE, White SA, Gelinas L, Strauss DH (2021). Fair payment and just benefits to enhance diversity in clinical research. J Clin Transl Sci.

[41] American Community Survey B16001: Language Spoken at Home 2019. United States Census Bureau.

[42] Trinh-Shevrin C, Islam NS, Rey MJ (2009). Asian American communities and health: context, research, policy, and action. 1st edition.

[43] Pearson SE, Taylor J, Hoare DJ, Patel P, Baguley DM (2019). Exploring the experiences of cancer patients with chemotherapy-induced ototoxicity: qualitative study using online health care forums. JMIR Cancer.

[44] Yi EG, Adamek ME (2021). Alzheimer's caregivers' experience with and perceptions of the Affordable Care Act: thematic analysis of online discussion forums. J Appl Gerontol.

[45] Horgan A, McCarthy G, Sweeney J (2013). An evaluation of an online peer support forum for university students with depressive symptoms. Arch Psychiatr Nurs.

[46] Im EO, Lee B, Chee W, Dormire S, Brown A (2010). A national multiethnic online forum study on menopausal symptom experience. Nurs Res.

[47] Ferrante JM, Friedman A, Shaw EK, Howard J, Cohen DJ, Shahidi L (2016). Lessons learned designing and using an online discussion forum for care coordinators in primary care. Qual Health Res.

[48] Hibbard JH, Greene J (2013). What the evidence shows about patient activation: better health outcomes and care experiences; fewer data on costs. Health Aff.

[49] Greene J, Hibbard JH, Sacks R, Overton V, Parrotta CD (2015). When patient activation levels change, health outcomes and costs change, too. Health Aff.

[50] (2022). Technical Overview of the AmeriSpeak® Panel NORC'S Probability-Based Household Panel. AmeriSpeak.

[51] Sanders JJ, Johnson KS, Cannady K, Paladino J, Ford DW, Block SD, Sterba KR (2019). From barriers to assets: rethinking factors impacting advance care planning for African Americans. Palliat Support Care.

