# Supplementary material for: Online Forums as a Tool for Broader Inclusion of Voices on Health Care Communication Experiences and Serious Illness Care: Mixed Methods Study
Source: J Med Internet Res. 2023 Dec 6;25:e48550. doi: 10.2196/48550 (PMC10733833; doi:10.2196/48550)
Supplement: Multimedia Appendix 1 [file jmir_v25i1e48550_app1.docx]

**2021 Serious Illness Care and Communication Consumer Survey**

**Massachusetts Coalition for Serious Illness Care**

| **Survey Instrument** | |
| --- | --- |
| Introduction: Today, AmeriSpeak is conducting a survey with people about their health care experiences and perspectives, including people with serious illnesses as well as future thoughts about serious illnesses for everyone else. When you hear the term serious illness, it means a disease or health issue that makes you feel sick enough that it’s increasingly hard to do your normal levels of work and activity (for example, conditions like cancer, heart disease or dementia). We want to learn about your experiences and include your opinions. All answers will be kept confidential and anonymous. | |
| **Questions/Answer Choices** | **Source/Notes** |
| **Section 0: Screening questions** |  |
| 1. Generally speaking, would you say your health is…  (Poor, Fair, Good, Very good, Excellent, Don’t know) | Source: KFF Q46, used in 2019 |
| 2. Have you ever been diagnosed with any of the following? Please check all that apply.  1 Diabetes  2 Asthma, lung disease, emphysema, or COPD  3 Heart disease or had a stroke  4 Cancer  5 Alzheimer’s disease, dementia or memory loss  6 Depression, anxiety or other serious mental health problems  7 Chronic kidney disease or kidney failure  8 None of these  3. Over the last 12 months, would you say that you have been feeling sicker and that it’s been getting harder to do your normal levels of work and activity?  (yes/no) | *Note: if yes to Q2 (any of 1-7) AND Q3, then the respondent falls into bucket of serious illness.* |
| 4. Do you have a disability?  (Yes/No) |  |
| 5. Do you currently help someone close to you **who has a lot of medical or health needs/conditions,** with one or more of the following?   - Make medical and health care decisions - Handle paperwork and bills related to healthcare - Provide physical care and assistance during the day or night - Coordinate care and transport to appointments   People who do these things for someone close to them are sometimes called caregivers.   1. Yes 2. No   5A. The next few questions will be about the person you care for as described in the previous question.  If you care for more than one person, please think of the **one person you spend the most time with**.  5B. How involved are you in the **medical care** of person you care for?  1 - Not at all involved  2 - Not very involved  3 - Fairly involved  4 - Very involved  5C. Does the person you care for live in a nursing home or assisted living facility?  (Yes/No)  5D. Does the person you care for have a serious illness? Remember a serious illness means a disease or health issue that makes a person feel sick enough that it’s increasingly hard to do their normal levels of work and activity, for example, conditions like cancer, heart disease or dementia).  (Yes/No) | Display 5A and Ask Q5B-D if Q5=Yes  If Q5B=3 or 4, classify as “Caregiver” |
| **Section 1A: Experiences of the health care system**  The next series of questions ask about your overall experiences with getting health care. When answering these questions, think about your experiences with health care visits in the last three years. |  |
| 6. Generally speaking, how much effort would you say doctors, nurses, and other health professionals make to help you understand your health issues?  (4-point Likert: No effort, A little effort, Some effort, A lot of effort) | Source: adapted from Elwyn’s CollaboRATE. |
| 7. Generally speaking, how much effort would you say doctors, nurses, and other health professionals make to listen to the things that matter most to you about your health issues?  (4-point Likert: No effort, A little effort, Some effort, A lot of effort) | Source: adapted from Elwyn’s CollaboRATE. |
| 8. Generally speaking, how much effort would you say doctors, nurses, and other health professionals make to include what matters most to you in making decisions about your care and treatments?  (4-point Likert: No effort, A little effort, Some effort, A lot of effort) | Source: adapted from Elwyn’s CollaboRATE. |
| 9. How often have you left a health care visit feeling unsure about your medicines, what was discussed, or what to do next?  (5-point Likert - Never/Rarely/Sometimes/Often/Always) |  |
| 10. Generally speaking, how well do you feel that doctors, nurses and other health professionals understand:   - Your life priorities - The activities that bring you joy and meaning - Your health goals - Your financial situation - The most important relationships in your life - Your faith or spirituality - Your culture   (4-point Likert: Not at all, Not very well, Fairly well, Very well, Don’t know) |  |
| 11. Do you have a specific doctor, nurse, or other health professional who understands you and what is important to you?  (Yes/No) |  |
| 12. How confident are you that you can control and manage most of your health problems?  N/A - I don’t have any health problems  Not very confident  Somewhat confident  Very confident |  |
| 13. How often have you been afraid to ask questions, speak up, or disagree with doctors, nurses, and other health professionals because you’re worried it may impact your care?  (4-point Likert - Almost none of the time/Some of the time/Most of the time/Almost all of the time/Don’t know) |  |
| 14. How much of the time do you trust doctors, nurses, and other health professionals to do what is right for you?  (4-point Likert - Almost none of the time/Some of the time/Most of the time/Almost all of the time/Don’t know) | Source: Adapted from KFF Undefeated Q3 |
| 15. How much of the time do doctors, nurses, and other health professionals treat you with dignity and respect?  (4-point Likert - Almost none of the time/Some of the time/Most of the time/Almost all of the time/Don’t know) | Source: Adapted from KFF Undefeated Q3 |
| 16. How often do doctors, nurses, and other health professionals talk down to you or make you feel inferior? (4-point Likert - Almost none of the time/Some of the time/Most of the time/Almost all of the time/Don’t know) | Loosely adapted from KFF/Undefeated Q30 |
| 17. Was there a time in the last 12 months when you felt that you were treated unfairly by doctors, nurses, or other health professionals?  (Yes/No) | Source: KFF |
| 18. (*Asked if “yes” to prior question*): What do you think are the main reasons for being treated unfairly? (Check all that apply)   - Because of my race or ethnicity - Because of the language I speak - Because of my age - Because of my gender - Because of my sexual orientation - Because of my income - Because of my disability - Because of my diagnosis or illness - Because of whether I have insurance - Other: (please specify) - Don’t know |  |
| **Section 1B: Caregiver’s Experience in healthcare system**  The next series of questions ask about your overall experiences helping the person you care for get health care. When answering these questions, think about your experiences with health care visits with the person you care for in the last three years. | Asked only to respondents who are caregivers. |
| 19. Generally speaking, how much effort would you say doctors, nurses, and other health professionals make to help you or the person you care for understand their health issues?  (4-point Likert: No effort, A little effort, Some effort, A lot of effort, Don’t know) | Source: adapted from Elwyn’s CollaboRATE. |
| 20. Generally speaking, how much effort would you say doctors, nurses, and other health professionals make to listen to the things that matter most to the person you care for?  (4-point Likert: No effort, A little effort, Some effort, A lot of effort, Don’t know) | Source: adapted from Elwyn’s CollaboRATE. |
| 21. Generally speaking, how much effort would you say doctors, nurses, and other health professionals make to include what matters most to the person you care for in making decisions about their care and treatments?  (4-point Likert: No effort, A little effort, Some effort, A lot of effort, Don’t know) | Source: adapted from Elwyn’s CollaboRATE. |
| 22. How often have you left a health care visit feeling unsure about the medicines, what was discussed, or what to do next for the person you care for?  (Likert: Never/Rarely/Sometimes/Often/Always, Don’t know) |  |
| 23. Generally speaking, how well do you feel that doctors, nurses, and other health professionals understand the following about the person you care for:   - Their life priorities - The activities that bring them joy and meaning - Their health goals - Their financial situation - The most important relationships in their life - Their faith or spirituality - Their culture   (4-point Likert Grid - Not at all, Not very well, Fairly well, Very well, Don’t know) |  |
| 24. Is there a specific health professional (doctor, nurse, or other health professional) who understands the priorities and what is important to the person you care for? (Yes/No/Don’t know) |  |
| 25. How confident are you that you can control and manage most of the health problems of the person you care for?   - N/A - Not very confident - Somewhat confident - Very confident |  |
| 26. How often have you been afraid to ask questions, speak up, or disagree with doctors, nurses, and other health professionals because you’re worried it may impact the care of the person you care for?  (4-point Likert - Almost none of the time/Some of the time/Most of the time/Almost all of the time/Don’t know) |  |
| 27. How much of the time do you think you can trust doctors, nurses, and other health care professionals to do what is right for the person you care for?  (4-point Likert - Almost none of the time/Some of the time/Most of the time/Almost all of the time/Don’t know) |  |
| 28. How much of the time do doctors, nurses, and other health professionals treat the person you care for with dignity and respect?  (4-point Likert - Almost none of the time/Some of the time/Most of the time/Almost all of the time/Don’t know) |  |
| 29 How often do doctors, nurses, and other health professionals talk down to the person you care for or make them feel inferior?  (4-point Likert - Almost none of the time/Some of the time/Most of the time/Almost all of the time/Don’t know) |  |
| 30. Was there a time in the last 12 months when you felt that the person you care for was treated unfairly by doctors, nurses, or other health professionals?  (Yes/No) |  |
| 31. (*Asked if “yes” to prior question*): What do you think are the main reasons for them being treated unfairly? (Check all that apply)   - Because of their race or ethnicity - Because of the language they speak - Because of their age - Because of their gender - Because of their sexual orientation - Because of their income - Because of their disability - Because of their diagnosis or illness - Because of whether they have insurance - Other: (please specify) - Don’t know |  |
| **Section 1c: General/System questions** | Do not display section break description. OK to start on new page, but no section transition needed. |
| 32. Generally speaking, how fair or unfair would you say the US health care system is?  (4-point Likert - Very unfair, Somewhat unfair, Somewhat fair, Very fair) |  |
| 33. How would you rate the US health care system when it comes to meeting the needs of people with serious illnesses?  (4-point Likert - Excellent/Good/Fair/Poor/Not sure) | Source: Adapted from KFF 2017 |
| 34. In your opinion, how much improvement is needed in the following areas to better meet the needs for people with serious illness?  (5-point Likert - No improvement, A little improvement, Some improvement, Quite a bit of improvement, A great deal of improvement, Don’t know)(Visually separate DK?)   1. Reducing cost of treatments and medicines 2. More support coordinating all the different doctors, appointments, and medicines 3. Helping with basic needs (housing, food, utilities and transportation) 4. More options for care at home 5. More support in people’s own language 6. Eliminating racism and discrimination 7. More access to the best treatment options 8. More support for mental health 9. More spiritual care support 10. More assistance with daily activities (dressing, meals, and household chores) 11. More assistance ensuring their home/residence/facility meets their needs (e.g. in terms of accessibility, mobility, safety) 12. More support for family members or friends who help take care of people with serious illnesses 13. More access to health insurance 14. Don’t know |  |
| 35. How important is it for doctors, nurses, or other health professionals to know about their patients’ priorities and what’s important to them?   1. Not at all important - They can provide high quality care without knowing this 2. Not very important 3. Fairly important 4. Very important - They cannot provide high quality care without knowing this |  |
| **Section 2: Personal Experience with Advance Care Planning (conversation, forms)**  Now we are going to ask you questions about planning for your future care, especially in moments when you are not able to speak for yourself and your thoughts about your current or a potential future serious illness. | Intention: to measure behavior. Can compare nationally to KFF 2017 data.  NOTE: Asked of all respondents; those with serious illness will be asked question with text in brackets omitted [if you become seriously ill] |
| 36. Have you ever had a serious conversation with a spouse, parent, child, or someone else close to you about your wishes for your medical care [if you become seriously ill], such as the types of treatments you do or don’t want to receive?  (Yes/No) | **Tracking Question**:  Source: KFF 2017 Q15 (Answer choices limited; selected in part based on large racial/ethnic disparities in responses) |
| 37. Have you ever had a serious conversation with a spouse, parent, child, or someone else close to you about who will make decisions about your medical care if you can no longer make them on your own?  (Yes/No) | **Tracking Question**:  Source: KFF 2017 Q15 (Answer choices limited; selected in part based on large racial/ethnic disparities in responses) |
| 38. Have you ever had a serious conversation with a doctor, nurse, or other health professional about your wishes for your medical care [if you become seriously ill], such as the types of treatments you do or don’t want to receive?  (Yes/No) | **Tracking Question**:  Source: KFF 2017 Q19 (Answer choices limited; selected based on large racial/ethnic disparities in responses) |
| [If no to 38]  39. Would you want to talk to a doctor, nurse, or other health professional about your wishes for your medical care [if you became seriously ill], such as the types of treatments you do and don’t want to receive?  (Yes/No) | **Tracking Question:**  Source: KFF 2017 Q22 |
| 40. Have you ever had a serious conversation with a doctor, nurse, or other health professional about who will make decisions about your medical care if you can no longer make them on your own?  (Yes/No) | **Tracking Question**:  Source: KFF 2017 Q19 (Answer choices limited; selected based on large racial/ethnic disparities in responses) |
| 41. Do you have a written document that:  (Grid: Yes/No/Don’t Know)   1. Names who you want to make decisions about your medical care if you can no longer make them on your own. 2. Describes your wishes for your medical care [if you become seriously ill], such as the types of treatments you do or don’t want to receive. | **Tracking Question:**  Source: KFF 2017 Q27 |
| 42. (Conditional on NO answer to 41**B**) There are different reasons why people may not have their wishes for medical care written down. Are any of the following a reason you have not written down your wishes for your medical care [if you became seriously ill]? Check all that apply.   1. There are too many other things to worry about right now. 2. You don’t want to think about sickness and death. 3. You haven’t thought about it (e.g. too young, too healthy). 4. You have never heard of it. 5. You want your doctors to make the decisions for you when needed. 6. You don’t have anyone that you can make responsible for decisions about your care. 7. You don’t know how to begin or would need help to do it. 8. Your family and people who make decisions will know what you want. 9. You don’t think these documents will make any difference in your care. 10. You are worried that having these documents will mean you get worse care. 11. This is not something that people in your culture, religion, or family do. 12. Other (please specify) | Sources: KFF 2017 Q44; MCSIC 2019, sourced from focus groups and collaborators in 2020 |
| 43. How worried are you about the following [if you were to become seriously ill]?  (4-point Likert: Not at all worried/Not too worried/Somewhat worried/Very worried)   1. You will have trouble affording the medical care or support services you need (e.g. for meals, household chores) 2. You will not be able to continue living where you want to. 3. Your family and other people who matter will struggle caring for you during your illness and after your death, for example with finances or their feelings. 4. You won’t get the best care because of your race, ethnicity, age, income, disability or other reason. 5. You may not have access to all of the best treatment options. 6. You won’t understand how to make the best choices for treatment. 7. You will have a lot of pain, stress, anxiety or depression. 8. If you can't speak for yourself, your family/friends won't make the best/right decisions about your care 9. You will have trouble managing all of your appointments, tests, medications, and instructions from your doctors. |  |
| 44. If you are seriously ill or were to become seriously ill, how important do you think each of the following are or would be in supporting you through your illness?  (5-point Likert: Not at all important/Not very important/Somewhat important/Very important/Extremely important)   1. Trust that the providers are taking care of you 2. Your family 3. Your friends 4. Your faith / Your faith community 5. Your spirituality 6. Staying physically active 7. Engaging in your hobbies 8. Finding purpose in your job, volunteering, activities in your home and community 9. Having someone help you navigate issues related to your illness |  |
| **Section 2b: Caregiver Experience with Advance Care Planning (conversation, forms)**  Now we’re going to ask you questions about planning future care for the person you care for, especially for moments when they are not able to speak for themselves, as well as your thoughts about their current or a potential future serious illness. If you care for more than one person, think of the person you spend the most time with. | Show this section only to “Caregiver” subgroup. |
| 45. Have you ever had a serious conversation about the person you care for with a doctor or other health care provider about their wishes for their medical care, such as the types of treatments they do or don’t want to receive? (Yes/No) | Source: Adapted KFF survey (answer choices limited) for caregiver. |
| 46. Have you ever had a serious conversation about the person you care for with a doctor or other health care provider about who will make decisions about their medical care if they can no longer make them on their own? (Yes/No) |  |
| 47. Does the person you care for have a written document that:  (Grid: Yes/No/Don’t know)   1. Names who they want to make decisions about their medical care if they can no longer make them on their own. 2. Describes their wishes for their medical care, such as the types of treatments they do or don’t want to receive. | Source: KFF 2017 Q77 |
| 48 (Conditional on NO answer on **Q47B)**. There are different reasons why people may not have their wishes for medical care written down. To your knowledge, are any of the following a reason the person you care for doesn’t have written down their wishes for their medical care? (Check all that apply)   1. There are too many other things to worry about right now. 2. They don’t want to think about sickness and death. 3. They haven’t thought about it (e.g., too young, too healthy) 4. They have never heard of it. 5. They want their doctors to make the decisions when needed. 6. They don’t know how to begin or would need help to do it. 7. Their family and people who make decisions will know what they want. 8. They don’t think these documents will make any difference in their care. 9. They are worried that having these documents will mean they get worse care. 10. This is not something that people in their culture, religion, or family do. 11. Don’t know. 12. Other (please specify). |  |
| 49. How worried are you about the following when it comes to the person you care for?  (4-point Likert: Not at all worried/Not too worried/Somewhat worried/Very worried)   1. The person you care for will have trouble affording the medical care or support services they need (e.g. for meals, household chores). 2. The person you care for will not be able to continue living where they want to. 3. You and others will struggle caring for them during their illness and after their death, for example with finances or your feelings. 4. The person you care for won’t get the best care because of their race, ethnicity, age, income, disability or other reason. 5. The person you care for may not have access to all of the best treatment options. 6. You or the person you care for won’t understand how to make the best choices for treatment. 7. The person you care for will have a lot of pain, stress, anxiety or depression. 8. If the person you care for can't speak for themselves, you won't make the best/right decisions about their care. 9. You will have trouble managing all of their appointments, tests, medications, and instructions from their doctors. | SOURCE: CAPC 2019, KFF 2017 Q12, other surveys |
| **Section 2c: Caregiver’s Patient Demographics.**  We have just a few more questions about the background and health of the person you care for. | Show this section only to “Caregiver” subgroup. |
| 50. What is the gender or gender identity of the person you care for?  1 Male  2 Female  3 Transgender Female  4 Transgender Male  5 Non-binary  6 Prefer not to answer/Don’t know  51. What is the age of the person you care for? (Your best estimate is fine if you’re not sure of their exact age.)  [ENTER WHOLE NUMBER]  52. What is the race of the person you care for? (Select all that apply)  1 White  2 Black, or African American  3 American Indian or Alaska Native  4 Asian  5 Some other Race  6 Prefer not to answer/Don’t know  53. Is the person you care for of Hispanic, Latino or Spanish origin?  1 No, not of Hispanic, Latino or Spanish origin  2 Yes, Hispanic, Latino, Spanish origin  3 Prefer not to answer/Don’t know  54. Is the person you care for deaf or do they have serious difficulty hearing? (Yes/No)  55. Is the person you care for blind or do they have serious difficulty seeing, even when wearing glasses? (Yes/No)  56. Because of a physical, mental, or emotional condition, does the person you care for have serious difficulty concentrating, remembering, or making decisions? (Yes/No)  57. Does the person you care for have serious difficulty walking or climbing stairs? (Yes/No)  58. Does the person you care for have difficulty dressing or bathing? (Yes/No)  59. Because of a physical, mental, or emotional condition, does the person you care for have difficulty doing errands alone such as visiting a doctor's office or shopping? (Yes/No)  60. Has the person you care for ever been diagnosed with COVID-19? (Yes/No)  61. How important is faith or spirituality in the life of the person you care for?  1 Very important  2 Somewhat important  3 Not too important  4 Not important at all  5 Prefer not to answer / Don’t know |  |
| **Section 3: Demographics** |  |
| 62. Have you or someone in your household ever been diagnosed with COVID-19? (Please check all that apply.)   1. You 2. Member(s) of your household 3. Neither of these |  |
| 63. Are you deaf or do you have serious difficulty hearing?  (Yes/No)  64. Are you blind or do you have serious difficulty seeing, even when wearing glasses?  (Yes/No)  65. Because of a physical, mental, or emotional condition, do you have serious difficulty concentrating, remembering, or making decisions?  (Yes/No)  66. Do you have serious difficulty walking or climbing stairs?  (Yes/No)  67. Do you have difficulty dressing or bathing?  (Yes/No)  68. Because of a physical, mental, or emotional condition, do you have difficulty doing errands alone such as visiting a doctor's office or shopping?  (Yes/No) | Source: Office of Minority Health disability questions (<https://minorityhealth.hhs.gov/omh/browse.aspx?lvl=3&lvlid=53>)  **VALIDATED Qs - cannot change** |
| 69. What is your gender or gender identity?  1 Male  2 Female  3 Transgender Female  4 Transgender Male  5 Non-binary | *Note: Asking this because NORC only gives us male/female binary.* |
| 70. Do you identify your race as...? (Select all that apply)  1 White  2 Black, or African American  3 American Indian or Alaska Native  4 Asian  5 Some other Race  6 Prefer not to answer |  |
| 71. Are you of Hispanic, Latino or Spanish origin?  1 No, not of Hispanic, Latino or Spanish origin  2 Yes, Hispanic, Latino, Spanish origin |  |
| 72. How important is faith or spirituality in your life?  1 Very important  2 Somewhat important  3 Not too important  4 Not important at all  5 Prefer not to answer |  |
