# Supplementary material for: Online Forums as a Tool for Broader Inclusion of Voices on Health Care Communication Experiences and Serious Illness Care: Mixed Methods Study
Source: J Med Internet Res. 2023 Dec 6;25:e48550. doi: 10.2196/48550 (PMC10733833; doi:10.2196/48550)
Supplement: Multimedia Appendix 2 [file jmir_v25i1e48550_app2.docx]

| **Objective** | **Activity Type** | **Questions** | **Comments/**  **Notes** |
| --- | --- | --- | --- |
| **WEEK 1** | | | |
| **Purpose and introductions** |  |  |  |
| Warm up, intros, expectation setting | Discussion board | ***Introduction – POST ON MONDAY 6/21 – 9AM***   - Hello and welcome! I’m Beth and I will be facilitating our conversations over the next 2 weeks. I’m looking forward to getting to know you all and appreciate you making time to share your candid thoughts and opinions. - **We’re here to help the Massachusetts Coalition for Serious Illness Care** – a nonprofit comprising 100+ organizations that in some way are involved in discussions concerning people’s health care. These member organizations represent a wide spectrum of health professionals/organizations/providers including – hospitals, doctors, nurses, counselors, health plans, social workers, attorneys, community groups, faith organizations and others. The Coalition also partners with other nonprofit organizations across the country. (more about the Coalition [here](http://maseriouscare.org/about)). - We’ll be spending most of our time **talking about health care experiences** - with a specific emphasis on serious illness care - whether that’s something you’ve experienced firsthand, know someone who has and/or have thoughts about if you were to face a serious illness in the future. For this research - **serious illness** is defined as, *a* *disease or health issue such as cancer, heart disease or dementia that makes you feel sick enough that it’s increasingly hard to do your normal levels of work and activity*. - **The Coalition recognizes that there is much room for improvement across the health care system** (whether that’s one on one interactions with your doctor or within bigger environments such as a hospital); their intent is to use **learnings from this research to help their member organizations** - **improve overall health care experiences.** - I want you to think of this community as a **safe space for honest conversation**. I know for a variety of reasons, talking about health care experiences can be stressful. You’ll see once we get started that I’ll be asking about *all* types of experiences - the good, the bad and the ugly (though will make sure that our conversations are productive and that we don’t spend our time in a downward spiral). - Hoping that’s enough context to get us started! Here’s what you can expect over the next 2 weeks: I will post **~ 7 different activities each week** (an activity could be a discussion that includes 3-4 open ended questions, a short survey or some other fun (and not time consuming) exercise. Expect to see emails from [**fginfo@itracks.com**](mailto:fginfo@itracks.com) announcing the new activities. **Your thoughtful and timely responses are so important.** - **Stories are key!**  Please keep that in mind when providing your responses. No need to share names or any other personal information – but to the extent you can **share scenarios and circumstances and the feelings you experienced** will make the learnings from this community even more relatable. - **I might come back to you with a follow up question** – asking you to elaborate on a thought to dig in deeper. You can also build on each other’s ideas and questions. - If at any point during the 2 weeks you have a question and/or are experiencing technical challenges in any way – **don’t hesitate to reach out to me or tech support at help@itracks.com.** - And lastly – don’t forget – if you provide thoughtful and timely responses to all of the activities in the next 2 weeks, **you’ll earn a $125 Amazon gift card!**   ***Icebreaker – POST ON MONDAY 6/21 – 9AM***  Let’s get started! We’ll begin with a quick icebreaker so that we can get to know one another. In a few sentences, tell me about a **community you feel especially connected to and why** (“community” could be your neighborhood, church, school, hobby, sport, etc.). I’ll start …   - For the last 20 years, I have been a member of my local YMCA. It’s about as plain and simple as you can get from a gym standpoint - but the people who work there - and my workout buddies have been an absolute lifeline for me, particularly over the last year and a half - my exercise class didn’t miss a beat despite the many Covid restrictions … moving from in person to Zoom, to outdoors in freezing temps … I am incredibly grateful for this community. | Throughout the two weeks …   - If needed, remind participants about how we are defining *doctor*, *serious illness*, *experiences*, etc. - When engaging in conversation about serious illness - make this feel more human … and frame as *living* with serious illness (vs. simply saying, people with serious illnesses). - Listen for the ways in which ***faith*** plays a role in overall health and wellbeing, navigating health care needs and experiences, etc. |
| **Overall health care experiences** |  |  |  |
|  | Discussion board  *Possible word cloud activity so that we can more visually see associations with health care experiences* | ***Activity 1 – POST ON MONDAY 6/21 – 9AM [AFTER ICEBREAKER]***  We’re going to talk a lot about **health care experiences** – whether it’s one on one with your doctor (**for simplicity’s sake - over the next 2 weeks, any time I say “doctor” - think of the health professional(s) you see most often which could entail a doctor, nurse practitioner, specialist, etc.)**, with your doctor’s office or community health center, or within bigger health care environments such as a clinic or hospital*.*   - Without overthinking it … what’s the first thing that comes to mind when you think of your health care experiences? Share that thought – and then tell me *why* you chose to share this one thing.   ***Keep in mind when you answer these questions ...*** (1) when I say “experiences”, focus on how you were treated and cared for and how you felt, and (2) when I say “they”, think of an individual such as your doctor or an entire system such as a hospital.   - FOLLOW UP WITH: As I mentioned in the intro - I’m interested in hearing about all types of health care experiences - the good, the bad and the ugly. Let’s start with something good … **share an example of an especially positive health care experience** (whether it was one on one with your doctor, your doctor’s office or a hospital). Is this something you experience often – or was this more a one-time thing? - AND THEN: Now **share an example of an especially negative health care experience** (whether it was one on one with your doctor, your doctor’s office, or a hospital). Is this something you experience often – or was this more a one-time thing? | Listen for instances when someone was being talked down to, stereotyping, racism. **Probe to understand these situations more fully.**  Listen for telehealth experiences and hospital at home (especially as it relates to coming out of covid and whether people living with serious illness are continuing to want complex/clinical care at home). |

|  | Discussion board | ***Activity 2 – POST ON MONDAY, 6/21 – 3PM***   - How – if at all - have your **health care experiences** (whether they’ve been one on one with your doctor, experiences you’ve had at your doctor’s office or within bigger health environments such as a clinic or hospital) **changed** (such as the ways in which your doctor engages you in conversation - the types of questions they ask you, the kind of information you want to share, the worries you may have and the extent to which your doctor and/or those you’re interacting with at a hospital help to put those worries at ease, the logistics associated with seeing different types of doctors and specialists, scheduling appointments, etc.) **as you have gotten older and/or have faced a serious illness**?   If you don’t feel as though you can truly comment here based on your age or health status - **share your perceptions** - that is, what you *think* could change if you were to face a serious illness OR share through the eyes of someone close to you who has gotten older and/or has faced a serious illness. How have their health care experiences changed, if at all?  ***And remember ...*** (1) when I say “experiences”, focus on how you were treated and cared for and how you felt, and (2) when I say “they”, think of an individual such as your doctor or an entire system such as a hospital. | Listen for conversations and experiences involving quality of life, palliative care, death; conversations with a doctor to discuss goals, values, preferences. |
| --- | --- | --- | --- |
|  | TBD | ***Activity 3 – POST ON TUESDAY, 6/22 – 9AM***   - Let’s dig into your health care experiences a bit more. Starting with the *positive* health care experiences (either with your doctor, your doctor’s office, clinic, hospital, etc.) – who and what primarily contributed to this positive experience(s)? What was it that they said or did that struck you as positive? And – how did these actions make you feel? - FOLLOW UP WITH: Now let’s focus on the *negative* health care experiences. Who or what primarily contributed to this negative experience(s)? What was it that they said or did that struck you as negative? And – how did these actions make you feel?   - Reflecting more on negative experiences - have you experienced any form of discrimination or bias and/or instances when it felt as though someone was talking down to you? If you are comfortable doing so - please share an experience. - AND THEN: to what extent - if at all - are these experiences (positive or negative) also happening to people you know? In other words - are the experiences happening more broadly or are they more isolated? | Listen for those true inflection points before, during and after visits with doctors and/or hospitals that have positive or negative impact on people.  Listen for frustrations related to transportation woes, traffic, parking; taking time out of the work day to see a specialist, etc. How can a doctor or system be more empathetic to these frustrations? What would make it easier for the individual?  Listen for ways participants have felt mistreated – racism/ bias, insurance coverage, guilt/shame, etc. |
|  | Brainstorm | ***Activity 4 – POST ON TUESDAY, 6/22 – 3PM***   - Reflecting on the negative health care experiences you’ve shared so far - which specific aspects of the health care system rise to the top as the most challenging … things you wish you didn’t have to experience when getting the care you need?   FOLLOW UP WITH: for those of you who have a serious illness (or care for someone with a serious illness) - do you have anything new or different to add here? | Cost will be a big pain point. Recognize this and push participants to share other pain point examples |
| **Empathy and trust** |  |  |  |
|  | TBD | ***Activity 5 – POST ON WEDNESDAY, 6/23 – 9AM***  We’ll start today by talking about specific moments in your health care experiences when you felt as though people had your best interests at heart ...when you felt validated, respected and/or treated with dignity.   - Share an example of when you felt validated, respected and/or treated with dignity (for example, you met one on one with your doctor and they gave you their full attention and made you feel comfortable - so much so that you shared how you were really feeling).   - What impact did this (feeling validated, respected and/or treated with dignity) have on you? For example, were you more willing to share information or ask more questions? - FOLLOW UP WITH: And conversely – share an example of when you did *not* feel validated, respected and/or treated with dignity.   - What impact did this (not feeling validated, respected and/or treated with dignity) have on you? For example, did you hold back on asking certain questions or sharing certain information, did you lean on other sources for the information you needed, etc.? | Possibly add a follow up question related to expectations vs. reality (things people may have read, watched on TV, heard via their community - the extent to which they bring this information to their doctor - and whether the doctor engages them in conversation, makes them feel heard, etc.)  Listen for instances when participants had to make decisions – and were afraid to ask questions.  Listen for ways the doctor shows *empathy* – particularly those who are also POC (and may feel like they too are victims of the system).  Listen for ways a hospital can better demonstrate validation and respect - such as staffing “navigators” to help coordinate next steps with specialists, be an advocate for people to lean on/complain to, etc. |
|  | TBD | ***Activity 6 - POST ON WEDNESDAY, 6/23 – 3PM***  In this activity, let’s focus on conversations you have with your doctor and what they may (or may not) be doing to get to know you, make you feel comfortable, etc.   - Have you ever had a **doctor who engages you in conversation about topics aside from those that are more medically related such as treatments, procedures, prescriptions, etc.**? For example - has your doctor asked about your relationships, community, culture or faith, finances, your job, etc.? - Whether you’ve had a doctor do this or not - how important is it to have these types of conversations with your doctor? Do you think doctors should know these things about you to give you the best care? - What specific things would you want to be asked/want them to know about you - and why? - What wouldn’t you want to be asked - and why?   POSSIBLY PROBE FURTHER:   - To what extent does your doctor make assumptions about who you are, your lifestyle, your preferences, etc.? And - what has been the impact of your doctor making these assumptions? - Reflect back on interactions with your doctor over the last year and whether they did or did not engage you in conversation about topics aside from those that are more medically related … What impact did these conversations (or the absence of these conversations) have on you? - Have you ever had a doctor who really knew you and worked with you to problem solve broader issues such as the cost of your health care and the impact of this cost on your finances, the inconveniences of scheduling appointments and juggling work, child care, etc.? - FOLLOW UP WITH: For those of you who have faced a serious illness (or know of someone who has), do you have anything new or different to add here? Do your expectations (specific to being asked about your life aside from the purpose of your visit, the illness you have, etc.) change in any way and how so?   - What specific things would you want to be asked - and why?   **Remember**: we’re defining **serious illness** as a disease or health issue such as cancer, heart disease or dementia that makes you feel sick enough that it’s increasingly hard to do your normal levels of work and activity. | Listen for reasons not to engage in conversation beyond the purpose of visit … sentiment such as, “If I’m honest – will that mean I’ll receive lesser quality care?”  Listen for instances of lowered power imbalances.  Listen for what feels ‘safe and not safe’ to share  Ultimately - what does it take to get to collaborative problem solving, mutual respect, working together to find the right solution for the individual? |

|  | TBD | ***Activity 7 – POST ON THURSDAY, 6/24 – 9AM***  Building on our conversations from earlier today - the extent to which your doctor takes time to get to know you and the overall impact this has on your health care - let’s now talk about **trust**.   - Share an experience you had with a doctor or hospital that negatively affected your trust in that doctor or hospital (these can be things you’ve experienced first hand and/or have seen happen to those you care for)?   - Did you at any point address your concerns with your doctor or someone at the hospital - and if so, what happened then?   - Have you experienced a loss of trust so significant that you wished you could change your doctor? - FOLLOW UP WITH: What about experiences that positively affected your trust in either a doctor or hospital? What was said or done specifically that made you want to trust that doctor or hospital?   **Remember** - we’re using “doctor” more broadly throughout our research; “doctor” can imply your doctor, your nurse practitioner, a specialist … the health professional(s) you see most often. | Trust builders vs. trust eroders …  Listen for: does lack of trust mean holding back information/ reluctance to be open/honest with their doctor?  Is *transparency a* factor of trust? For example - you may not feel as though a doctor is sharing all care/ treatment options because he/she knows that you can’t afford it.  Here again - listen for discrimination and bias of any type (consider directly asking if this doesn’t come up organically in conversation - the stigma behind disability, race, age, etc.) |
| --- | --- | --- | --- |

| **Quality care** |  |  |  |
| --- | --- | --- | --- |
|  | Brainstorm + discussion | ***Activity 8 – POST ON THURSDAY, 6/24 – 3PM***  We’ve covered a lot of ground this week! Let’s end this week with a bigger wrap up question … **What does good quality care look like to you**?  FOLLOW UP WITH: How, if at all, does the way in which you think about good *quality care* change if you factor in living with a serious illness (whether this is something you’ve experienced firsthand, can imagine facing or know someone who has)?  ***Activity 9– POST ON FRIDAY, 6/25 – 9AM***  I’ve actually got one last question this week – thank you again for your commitment to this community and providing such rich feedback.  Reflect back on the many topics we’ve covered this week – examples of when you felt respected, validated and/or treated with dignity; conversations you’re having with your doctor and what truly constitutes meaningful dialog; how your doctor earns your trust … and now, **think about what would matter most to you if you were to have a “serious illness conversation” with your doctor. What would you want them to say or do (or *not* say or do) in this type of conversation?**  For this activity, I’m defining “serious illness conversation” as a conversation a person has with their doctor when their health and wellbeing are declining and/or treatments are becoming less effective.  If you have a story/example to share that brings to life this type of conversation and things a doctor said or did that struck you as either positive or negative - please share. The context is super helpful.  PROBE IF NEEDED: is your doctor asking you about certain treatments such as CPR or if you want a DNR in place? Or if you want to name a person to speak on your behalf if you reach a point when you are unable to speak for yourself? At what point does this conversation take place? Who else should be included in this conversation? | Remind participants if needed - words/ language are fine here vs. full sentences. |
| **WEEK 2** | | | |
| **Language/ concept testing** |  |  |  |
|  | High lighting exercise - part 1 | ***Activity 10 – POST ON MONDAY, 6/28 – 9AM***  Congrats on getting through week 1! Love the thoughts and ideas you’ve shared so far. Let’s dig into week 2.  **At this point in our research, we are going to focus our time on getting your thoughts and reactions to ideas and concepts … understanding what resonates with you, what’s believable, what you could envision experiencing during your visits with your doctor and/or hospital.**  To get into the right mindset for these next few activities, imagine that you are having a conversation with your doctor and you are discussing options and choices for your health care (whether that’s related to a serious illness, like cancer, heart disease or dementia or routine management of a health condition like asthma or diabetes). What would you want your doctor to say or not say that would help you make decisions about what to do next for your current health care (or care you can imagine getting)?   - **STEP 1:** Read through six different statements (think of these as conversation prompts that your doctor would use to engage you in a conversation). - **STEP 2:**  Use a highlighting tool that includes 3 different colors (green = positive, yellow = neutral, red = negative) to indicate things you like, dislike and/or feel indifferent about within each statement (could be the words and/or whole phrases). - **STEP 3:**  Add comments that explain *why* you indicated what you did. ***The comments are super important - please take the time to be as clear as you can.***   **ITRACKS PROGRAMMING NOTES:** LINK TO ACTUAL HIGHLIGHTING EXERCISE AND RANDOMIZE STATEMENTS FOR EACH PARTICIPANT TO ELIMINATE BIAS.  **CONVERSATION STATEMENTS - FIRST ROUND OF 6:**  **I’m keeping up with the latest research, treatments and ideas**. Science is changing all the time, but I am an expert in your particular condition and am aware of up-to-date best practices and all the choices for your treatment. And if I don’t know, I will ask my colleagues. If it turns out we don’t offer one of those treatments or approaches here, we can talk about how to get it to you.  **We’ll figure this out together.** I’ll help you navigate through the choices ahead. We can talk about each one. I will make sure you really understand how each option will help you and what side effects or downsides it might have. Different choices may impact what you can do and how you’ll feel. Everyone is different in how they think about what a good quality of life means. You’re the expert on what’s right for you, so the more you share, the more I can support you. First, so that I can help you choose care that’s right for you, I need you to help me understand who and what really matters to you—your favorite activities, the people you love, your faith and culture.  **I welcome all of your ideas and questions;** If you hear or read about treatments from family, magazines or on the internet, or have ideas about alternative therapies, please share them with me. I won’t always know about everything, but I will listen and figure out how we can incorporate your ideas into our decisions. Any ideas and questions you bring to our discussion can help me do my best job for you.  **If you have any concerns, let me know.** If something ever doesn’t seem right, or you see an error in your records or plans or think there is something wrong with your treatment, it’s my and my team’s responsibility to fix it. It’s not your job to track all this stuff down.  **I know that health care and treatments can be expensive**. I wish that costs didn’t need to be involved in making care choices. I will offer all the choices I think may help your condition, but some might only help a little bit, some might not help at all and some may cost you a lot of money. We’ll take time to consider the options as we figure out what works best for you and support what matters most to you. I can’t provide you with the best quality care unless we’re working together.  **I get that, sometimes, life and other things can get in the way of your ability to get care.** If it’s tough for you to make the time for visits or afford the transportation costs to get here or another center involved in your care, let’s talk about it. There could be other options closer to home or we could even consider virtual options. Or if you have other worries such as immigration status, let me know and I will try to help. | Throughout week 2 - to what degree are we seeing participants express their frustration with the health care system and perhaps (in their own words) liken it to a transactional marketplace? Draw out from participants their thoughts and ideas on realistic change - specific examples of change that could positively impact their overall health and wellbeing. |
|  | High lighting exercise - part 2 | ***Activity 11 – POST ON MONDAY, 6/28 – 3PM***    We’re going to keep thinking about words and language.  Imagine again that you are having a conversation with your doctor and discussing options and choices for your health care (whether that’s related to a serious illness, like cancer, heart disease or dementia or routine management of a health condition like asthma or diabetes). What would you want your doctor to say or not say that would help you make decisions about what to do next for your current health care (or care you can imagine getting)?   - **STEP 1**: Read through six more statements (again, think of these as conversation prompts that your doctor would use to engage you in a conversation) - **STEP 2:** Use the highlighting tool to indicate things you like, dislike and/or feel indifferent about within each statement (could be the words and/or whole phrases). - **STEP 3**: Add comments that explain *why* you indicated what you did. ***Remember, the comments are super important - please take the time to be as clear as you can.***   **ITRACKS PROGRAMMING NOTES:**  LINK TO ACTUAL HIGHLIGHTING EXERCISE, RANDOMIZE STATEMENTS FOR EACH PARTICIPANT TO ELIMINATE BIAS AND MAKE SURE PARTICIPANTS DO ACTIVITY 9 BEFORE MOVING ONTO ACTIVITY 10.  **CONVERSATION STATEMENTS - SECOND ROUND OF 6:**  **I treat all my patients equally, and recommend the kind of care I would recommend for family or close friends**. People often ask me what I’d do if I myself, or my own mother, were the patient in their shoes. And I tell them it would be the same process: I take the time to explain how I would help my mom make decisions, based on what matters to her, her circumstances and priorities—just as I will do for you.  **Treatments only work if they work for you**. If you have too many pills to keep track of, need to run to different pharmacies to get them, or if appointments are too hard to fit into your life and it’s too hard to come see me or your other doctors, let me know. I may not always be able to figure it out right away, but I will always listen and try to problem-solve with you. If I know about challenges, I can do better.  **Someone—me myself, or a member of my team—will always call you back**. Your questions and concerns matter to me and my team, so you never have to worry that they will go unanswered or unaddressed.  **Your time matters to me**. If I am late for our appointment, I will try to make it up. As much as possible, I will try to make sure that all your appointments are at the same time, on the same day. Or if it works better for you, we can try and use virtual visits or home visits so you don’t have to travel to meet me in my office.  **Many patients are concerned that if they talk about challenges with health care costs, I will assume they can’t afford treatments.** Even if you share concerns about treatment costs, I will not withhold choices that can help you. And then if costs are a challenge, we can work together to figure out what is best for you. That can mean using lower cost treatment options or I can connect you to our financial counselor to talk about how to deal with all the medical bills.  **Let’s make a plan for your care in case you can’t speak for yourself one day.** We can’t plan for everything. But we can talk about what matters to you and what you’d want most if you couldn’t make your own decisions. We may not be able to predict every choice that would need making, but you can give those you love the guiding principles to confidently make decisions for you if they have to. So let’s talk about your values and preferences, and the care that’s right for you. Conversations about things we can’t control can actually help to give us a sense of control. |  |
|  | Ranking survey | ***Activity 12 - POST ON MONDAY, 6/28 - 3PM***  Now that you’ve provided feedback on the different types of conversation statements (prompts you could imagine your doctor using to engage you in a conversation) … I’d like you to review all 12 statements and:   1. **Pick the statement that matters *most* to you** - in terms of what you’d want your doctor to include in a conversation with you about your health care (and share why). 2. And then, **pick the statement that matters *least*** (and share why).   **ITRACKS PROGRAMMING NOTES:**  RANDOMIZE STATEMENTS BY PARTICIPANT TO ELIMINATE BIAS AND MAKE SURE PARTICIPANTS DO ACTIVITY 10 BEFORE MOVING ONTO ACTIVITY 11.  **CONVERSATION STATEMENTS - FULL SET OF 12:**  **I’m keeping up with the latest research, treatments, and ideas**. Science is changing all the time, but I am an expert in your particular condition and am aware of up-to-date best practices and all the choices for your treatment. And if I don’t know, I will ask my colleagues. If it turns out we don’t offer one of those treatments or approaches here, we can talk about how to get it to you.  **We’ll figure this out together.** I’ll help you navigate through the choices ahead. We can talk about each one. I will make sure you really understand how each option will help you and what side effects or downsides it might have. Different choices may impact what you can do and how you’ll feel. Everyone is different in how they think about what a good quality of life means. You’re the expert on what’s right for you, so the more you share, the more I can support you. First, so that I can help you choose care that’s right for you, I need you to help me understand who and what really matters to you—your favorite activities, the people you love, your faith and culture.  **I welcome all of your ideas and questions;** If you hear or read about treatments from family, magazines or on the internet, or have ideas about alternative therapies, please share them with me. I won’t always know about everything, but I will listen and figure out how we can incorporate your ideas into our decisions. Any ideas and questions you bring to our discussion can help me do my best job for you.  **If you have any concerns, let me know.** If something ever doesn’t seem right, or you see an error in your records or plans or think there is something wrong with your treatment, it’s my and my team’s responsibility to fix it. It’s not your job to track all this stuff down.  **I know that health care and treatments can be expensive**. I wish that costs didn’t need to be involved in making care choices. I will offer all the choices I think may help your condition, but some might only help a little bit, some might not help at all and some may cost you a lot of money. We’ll take time to consider the options as we figure out what works best for you and support what matters most to you. I can’t provide you with the best quality care unless we’re working together.  **I get that, sometimes, life and other things can get in the way of your ability to get care.** If it’s tough for you to make the time for visits or afford the transportation costs to get here or another center involved in your care, let’s talk about it. There could be other options closer to home or we could even consider virtual options. Or if you have other worries such as immigration status, let me know and I will try to help.  **I treat all my patients equally, and recommend the kind of care I would recommend for family or close friends**. People often ask me what I’d do if I myself, or my own mother, were the patient in their shoes. And I tell them it would be the same process: I take the time to explain how I would help my mom make decisions, based on what matters to her, her circumstances and priorities—just as I will do for you.  **Treatments only work if they work for you**. If you have too many pills to keep track of, need to run to different pharmacies to get them, or if appointments are too hard to fit into your life and it’s too hard to come see me or your other doctors, let me know. I may not always be able to figure it out right away, but I will always listen and try to problem-solve with you. If I know about challenges, I can do better.  **Someone—me myself, or a member of my team—will always call you back**. Your questions and concerns matter to me and my team, so you never have to worry that they will go unanswered or unaddressed.  **Your time matters to me**. If I am late for our appointment, I will try to make it up. As much as possible, I will try to make sure that all your appointments are at the same time, on the same day. Or if it works better for you, we can try and use virtual visits or home visits so you don’t have to travel to meet me in my office.  **Many patients are concerned that if they talk about challenges with health care costs, I will assume they can’t afford treatments.** Even if you share concerns about treatment costs, I will not withhold choices that can help you. And then if costs are a challenge, we can work together to figure out what is best for you. That can mean using lower cost treatment options or I can connect you to our financial counselor to talk about how to deal with all the medical bills.  **Let’s make a plan for your care in case you can’t speak for yourself one day.** We can’t plan for everything. But we can talk about what matters to you and what you’d want most if you couldn’t make your own decisions. We may not be able to predict every choice that would need making, but you can give those you love the guiding principles to confidently make decisions for you if they have to. So let’s talk about your values and preferences, and the care that’s right for you. Conversations about things we can’t control can actually help to give us a sense of control. |  |
|  | Rating survey | ***Activity 13 - POST ON TUESDAY, 6/29 - 9AM***  Imagine again that you are having a conversation with your doctor and discussing options and choices for your health care (whether that’s related to a serious illness, like cancer, heart disease or dementia or routine management of a health condition like asthma or diabetes). **To what extent would you feel comfortable answering the following questions (questions your doctor would ask you during a conversation about your health care)?**  For each question, indicate your level of comfort by rating on a scale of 1 to 5 where 1 = very comfortable and 5=not at all comfortable.  **CURRENT DIRECTIVE TEST QUESTIONS:**   1. Do you want to tell me about any bad health care experiences I should know about to better take care of you, such as things that happened in the past that you don’t want to happen again? 2. Before we focus on your condition, can I ask about what else is going on in your life—home, family, work—that could have an effect on your health or wellbeing? 3. In order to help give you the best care, I need you to help me understand who and what really matters to you. Could we take a few minutes to explore this? What are your life priorities right now? What are your favorite activities and what brings you joy? Who are the people you love? What should I know about your faith and culture? 4. Are there any concerns you would like to talk about? 5. What do you hope your health care can do for you? 6. Do you have a spouse, parent, child, or someone else close to you who can speak on your behalf if you can’t make your own health care decisions? 7. Are you worried about medical costs and affording care?   FOLLOW UP WITH OPEN-ENDED DISCUSSIONS - (1): is there anything missing from this list (other types of questions you think your doctor should be asking in conversations with you about your health care)?  AND THEN - (2): Is there anything more your doctor could say or do to make you feel more comfortable in these types of conversations about your health care?  **ITRACKS PROGRAMMING NOTES:** MAKE LIST OF QUESTIONS FROM RATING SURVEY VIEWABLE TO HELP PARTICIPANTS THINK THROUGH THESE OPEN ENDED QUESTIONS. ALSO MAKE SURE PARTICIPANTS CAN’T RESPOND TO THESE FOLLOW UP OPEN-ENDED QUESTIONS UNTIL THEY COMPLETE ACTIVITY 12 RATING SURVEY. |  |
|  |  | ***INTENTIONALLY NOT FIELDING AN ACTIVITY TUESDAY AFTERNOON GIVEN HEAVY ASKS ON MONDAY (ALL DAY) AND TUESDAY AM. WILL USE THIS TIME TO PROBE ON INPUT PROVIDED MONDAY - TUESDAY AM.*** |  |
|  | High lighting exercise | ***Activity 14 - POST ON WEDNESDAY, 6/30 – 9AM***  For this activity, we’re going to focus on **resources people can use to help get the health care and support they need.**  Imagine again that you are having a conversation with your doctor and you are discussing options and choices for your health care (whether that’s related to a serious illness, like cancer, heart disease or dementia or routine management of a health condition like asthma or diabetes). As part of this conversation, your doctor shares with you a resource designed to help you get the care and support you need.   - **STEP 1:**  Read through each resource (there are five total). - **STEP 2:** Use the highlighting tool to indicate what’s especially relevant/helpful to you and what is not. - **STEP 3:** Add comments that explain *why* you indicated what you did. ***Remember, the comments are super important - please take the time to be as clear as you can.***     **iTRACKS PROGRAMMING NOTES:** NAME OF RESOURCE IN PARENTHESES IS FOR INTERNAL PURPOSES - DO NOT SHOW TO PARTICIPANTS. RANDOMIZE THE FIVE RESOURCES FOR EACH PARTICIPANT TO ELIMINATE BIAS.  **RESOURCES - FULL SET OF FIVE.**  **(Palliative Care Description)**  A specially-trained team of doctors, nurses and other specialists at the hospital or clinic who work together with a patient’s other doctors to provide an extra layer of support for people living with serious illness. The care is focused on providing relief from the symptoms and stress of a serious illness. The team also helps to coordinate and communicate with all the patient's other doctors on what to expect and how to prepare for it. The goal is to improve quality of life for both the patient and the family. The care is also based on the needs of the person, not on the persons’ prognosis. It is appropriate at any age and at any stage in a serious illness, and it can be provided along with curative treatment.  **(Amalgamation of ideas Description)**  A person who works for a hospital or clinic and is assigned to help you advocate for yourself—to support you in solving any problems or fixing errors that may be made. For example, if you have questions you don’t feel you could ask at your appointment, or if errors in your medical record or billing statements were found, this person is there to help. Another example: If you were not comfortable with your doctor for some reason, or wanted to consider finding a new doctor, you could talk to this person about working with the hospital or clinic to find a solution.  **(PAF Description)**  A person who works for a nonprofit organization who is there to help you navigate any issues you face in accessing or affording your health care. They can gather information so you understand your insurance coverage, all the benefits you are eligible for, what costs (co-pay, premiums, deductibles, etc.) you are responsible for, and what other supports (charity care, drug discounts, negotiated rates, etc.) you may qualify for. Beyond payment, this person can also help you advocate for yourself with health caregivers and identify any additional needs—such as transportation, housing, food, and emotional support—you may have that could support your health and wellness.  **(ACCA Description)**  Trained care navigators from a faith-based program in your community that help individuals with serious illnesses and their caregivers identify and address their needs, access trusted resources, and empower people with tools and training. The care navigators come to your home several times over many months, working with you on many things: for example, connecting you with services that deliver healthy food or prepared meals; empowering you to prepare questions and concerns you want to raise with your doctors about your health, medications, and treatment; providing prayer or meditation training, respecting your spirituality; helping you complete advance care planning documents, such as a health care proxy, advance directive, or other forms that document your wishes for care; and providing support for your caregiver’s wellbeing as well as helping them learn how to better care for you.  **(Age-Friendly Health System/Patient Priorities Care description)**  A hospital or clinic where all the doctors and other clinicians and staff have special training, programs and time to make sure that everyone really understands you and what is important to you so they can make sure your health care supports your priorities and goals. For example, it could be taking additional time in each visit –no matter what you’ve come in for- to talk about what matters to you and what is going on in all parts of your life and including that information in your medical record so everyone can see it; then talking about what is working when it comes to your health care and what is not and working together with your doctor and the whole team to make sure your health goals are being met. | Possibly pull from quant survey  Where does ACP language fit within the hierarchy of needs?  Zero in on believability, resonance |
|  | Ranking survey |  |  |
|  | Rating survey | ***Activity 16 - POST ON WEDNESDAY, 6/30 – 3PM***  For this next activity, I’d like your reactions to what you believe constitutes ***good*** ***quality care***.   - **STEP 1:**  Read through each quality care statement (there are four total). The language in each statement may seem familiar as it reflects your thoughtful input from last week. - **STEP 2:** Use the three highlighter colors to indicate **what resonates with you** (green - positive), **what you are indifferent about** (yellow - neutral) and **what does not resonate with you** (red - negative) … could be the words and/or whole phrases. - **STEP 3:** Add comments that explain *why* you indicated what you did. ***Remember, the comments are super important - please take the time to be as clear as you can.***       **ITRACKS PROGRAMMING NOTES -** RANDOMIZE THE 4 QUALITY CARE STATEMENTS BY PARTICIPANT TO ELIMINATE BIAS AND MAKE SURE PARTICIPANTS DO ACTIVITY 16 BEFORE MOVING ONTO ACTIVITY 17.  **1. Good quality care is timely, convenient, and coordinated**: good quality means being able to see a doctor as soon as I need; it means teams of doctors work together and talk to each other, so I don’t have to update everyone when something changes; visits and tests are scheduled around me and my life, like using many more virtual/telehealth visits whenever possible and then being on time when I do have to come in; outreach and follow-up is proactive to make sure I get everything on schedule; doctors read my chart and information so I don’t have to repeat everything each time. Calls or online messages are returned quickly; refills and referrals are done without hassle, and results reported fast.  **2. Good quality care is from competent, knowledgeable doctors who know all the most effective treatments available**. Doctors will keep up with the latest research and make me feel confident in their knowledge by, for example, answering questions about their expertise and giving me straight answers to my questions. While everyone is human, quality care has minimal mistakes. But it also means admitting when they do not know something and being honest about issues.  **3. Good quality care is kind, empathetic, patient, non-judgmental, treating me (and everyone) as important and worthy of care**: everyone – from the doctors to the office staff – really listens to me, believes me, treats me with dignity, respect, recognizing that I know myself better than anyone and not assuming anything about me. They work to really understand me, treat me like a whole person, not as a number, recognizing the big picture, including how I am feeling emotionally as well as my physical symptoms. It’s really truly caring.  **4. Good quality care is collaborative with me**: it requires taking the time to learn about me so that my care is the best possible fit for me and to learn what is really going on – not just the symptoms but the root cause. It also means taking the time to help me understand what needs to be done and why, and allows me to weigh the risks and rewards of different courses of treatment, including honest considerations of cost. It means coming up with a plan together that works for me and my life. Good quality care means a relationship where doctors and patients are working together, trusting each other, and committed to my wellbeing.  ***Activity 17 – POST ON WEDNESDAY, 6/30 – 3PM***    Now that you’ve provided feedback on the good quality care statements, review all four again and pick the one that resonates ***MOST*** (and share why) and then pick the one that resonates ***LEAST*** (and share why).    **ITRACKS PROGRAMMING NOTES:** RANDOMIZE THE 4 QUALITY CARE STATEMENTS WITHIN THE SURVEY TO ELIMINATE BIAS AND MAKE SURE PARTICIPANTS DO ACTIVITY 16 BEFORE MOVING ONTO ACTIVITY 17.    **QUALITY CARE STATEMENTS - FULL SET OF FOUR**    **1. Good quality care is timely, convenient, and coordinated**: good quality means being able to see a doctor as soon as I need; it means teams of doctors work together and talk to each other, so I don’t have to update everyone when something changes; visits and tests are scheduled around me and my life, like using many more virtual/telehealth visits whenever possible and then being on time when I do have to come in; outreach and follow-up is proactive to make sure I get everything on schedule; doctors read my chart and information so I don’t have to repeat everything each time. Calls or online messages are returned quickly; refills and referrals are done without hassle, and results reported fast.    **2. Good quality care is from competent, knowledgeable doctors who know all the most effective treatments available**. Doctors will keep up with the latest research and make me feel confident in their knowledge by, for example, answering questions about their expertise and giving me straight answers to my questions. While everyone is human, quality care has minimal mistakes. But it also means admitting when they do not know something and being honest about issues.    **3. Good quality care is kind, empathetic, patient, non-judgmental, treating me (and everyone) as important and worthy of care**: everyone – from the doctors to the office staff – really listens to me, believes me, treats me with dignity, respect, recognizing that I know myself better than anyone and not assuming anything about me. They work to really understand me, treat me like a whole person, not as a number, recognizing the big picture, including how I am feeling emotionally as well as my physical symptoms. It’s really truly caring.    **4. Good quality care is collaborative with me**: it requires taking the time to learn about me so that my care is the best possible fit for me and to learn what is really going on – not just the symptoms but the root cause. It also means taking the time to help me understand what needs to be done and why, and allows me to weigh the risks and rewards of different courses of treatment, including honest considerations of cost. It means coming up with a plan together that works for me and my life. Good quality care means a relationship where doctors and patients are working together, trusting each other, and committed to my wellbeing. | For the quality statements - incorporate actual participant language (such as the positive and/or ideal health care experiences) from week 1. Test for believability/ resonance.  Consider looking at nuances by race, age, disability, serious illness, caregiver - on the backend. |

| **Wrap up** |  |  |  |
| --- | --- | --- | --- |
|  | Discussion board | ***Final activity – POST ON THURSDAY, 7/1 – 9AM***  Well – it’s hard to believe **we’re at the end of our 2 weeks already!** The ground we’ve covered is amazing – and your stories, opinions and ideas – are impressive and clearly come from your heart. Our last question:   - What **advice do you have for the Massachusetts Coalition for Serious Illness Care** as they continue to work with doctors (nurse practitioners, specialists, and other health professionals) and the environments in which they work (offices, clinics and hospitals) to create positive health care experiences?   **Thank you again for the time and effort you’ve put into this exploration over the last 2 weeks. On behalf of the Massachusetts Coalition for Serious Illness Care – I’m so grateful for your candor and your stories!** |  |
