# Supplementary material for: Online Forums as a Tool for Broader Inclusion of Voices on Health Care Communication Experiences and Serious Illness Care: Mixed Methods Study
Source: J Med Internet Res. 2023 Dec 6;25:e48550. doi: 10.2196/48550 (PMC10733833; doi:10.2196/48550)
Supplement: Multimedia Appendix 3 [file jmir_v25i1e48550_app3.docx]

**Research approach:**  we’ll create three separate communities comprised of 75 participants each, reflecting desired quota percentages across the 6 sub groups (1: Low income - defined as <$50K HHI + Black/African-American, 2: Low income - defined as <$50K HHI + Hispanic/Latinx , 3: People with disabilities, 4: People who have or have had a serious illness, 5: Older adults ages 65+, 6: Caregivers). Each community (referred to as Community A, Community B and Community C) will be tasked with reviewing and critiquing unique calls to action messaging. More context on the actual structure of the message testing is provided in activities 4-7.

| **Objective** | **Activity Type** | **Questions** | **Comments/**  **Notes** |
| --- | --- | --- | --- |
| **Purpose and introductions** |  |  |  |
| Warm up, intros, expectation setting | Discussion board | ***Introduction – POST ON MONDAY 8/9 – 9AM***   - Hello and welcome! I’m Beth and I will be facilitating our conversations this week. I’m looking forward to getting to know you all and appreciate you making time to share your candid thoughts and opinions. - **We’re here to help the Massachusetts Coalition for Serious Illness Care** – a nonprofit comprising 100+ organizations that in some way are involved in discussions concerning people’s health care. These member organizations represent a wide spectrum of health professionals, organizations and providers including – hospitals, doctors, nurses, counselors, health plans, social workers, attorneys, community groups, faith organizations and others. The Coalition also partners with other nonprofit organizations across the country (more about the Coalition [here](http://maseriouscare.org/about)). - We’ll be spending our time **sharing thoughts on health care experiences** - whether it’s one on one with your doctor, or visits to a community health center, hospital or clinic. **The Coalition recognizes that there is much room for improvement across the health care system**; their intent is to use **learnings from this research to help their member organizations improve overall health care experiences.** - Hoping that’s enough context to get us started! Here’s what you can expect this week: I will post **~ 8 different activities** (an activity could be a discussion that includes open ended questions, a short survey or some other fun (and not time consuming) exercise. Expect to see emails from [**fginfo@itracks.com**](mailto:fginfo@itracks.com) announcing the new activities. **Your thoughtful and timely responses are so important.** - **Stories are key!**  Please keep that in mind when providing your responses. No need to share names or any other personal information – but to the extent you can **share scenarios and circumstances and the feelings you experienced** will make the learnings from this community even more relatable. - **I might come back to you with a follow up question** – asking you to elaborate on a thought to dig in deeper. - If at any point during the week you have a question and/or are experiencing technical challenges in any way – **don’t hesitate to reach out to me or tech support at help@itracks.com.** - And lastly – don’t forget – if you provide thoughtful and timely responses to all of the activities posted this week, **you’ll earn a $100 Amazon gift card!**   ***Icebreaker – POST ON MONDAY 8/9 – 9AM***  Let’s get started! We’ll begin with a quick icebreaker so that we can get to know one another.  **What’s one health and/or wellness mantra you try to live by?**  I’ll start … I am a big believer in doing some form of exercise every day – whether it’s a fitness class, a bike ride or a walk around my neighborhood. This daily commitment has a huge impact on my mental health and puts me in the right frame of mind to tackle whatever’s on my plate. |  |
| **Follow ups to quant** |  |  |  |
|  | Survey + open ended “why” comments | ***Activity 1 – POST ON MONDAY 8/9 – 9AM [AFTER ICEBREAKER]***  We’re going to start with a short survey that addresses the degree to which your doctors and other health professionals understand what matters to you. **You’ll see after the survey that I’ve included space for you to share *why* you indicated what you did** - specifically, how did they learn this about you?  Generally speaking, how well do you feel that doctors, nurses and other health professionals understand:   - Your life priorities - The activities that bring you joy and meaning - Your health goals - Your financial situation - The most important relationships in your life - Your faith or spirituality - Your culture   **iTRACKS PROGRAMMING NOTES**:   - FOR EACH BULLET ABOVE, INCLUDE THE FOLLOWING 4-POINT SCALE: 1=Not at all, 2=Not very well, 3=Fairly well, 4=Very well - RANDOMIZE OPTIONS TO AVOID BIAS. - INCLUDE A FOLLOW-UP, OPEN-ENDED QUESTION FOR PARTICIPANTS TO ELABORATE ON WHY THEY INDICATED WHAT THEY DID. | Very light touch on probes; we’re trying to avoid biasing them towards starting to ‘realize’ their doc doesn’t really know them. We just want context for the qual results we got. I recognize that even asking them to explain why will probably make them a ‘harder grader’.  If needed – follow up with a separate probe that encourages people to dig deeper on whether their doctor understands what matters to them (and how the doctor surfaced this information – what was the context, in what ways did he/she ask the questions, make the individual feel comfortable, explain why he/she needed this information, etc.). |

|  | Survey + open ended “why” comments | ***Activity 2 – POST ON MONDAY, 8/9 – 3PM***    Following up on this morning’s activity about doctors, nurses and other health professionals understanding what matters to you … **how important is it that they know about your priorities and what’s important to you?** Tell me *why* you feel this way in the space below.   1. Not at all important - They can provide high quality care without knowing this 2. Not very important 3. Fairly important 4. Very important - They cannot provide high quality care without knowing this   **iTRACKS PROGRAMMING NOTES**: INCLUDE A FOLLOW-UP, OPEN-ENDED QUESTION FOR PARTICIPANTS TO ELABORATE ON WHY THEY CHOSE WHAT THEY DID. |  |
| --- | --- | --- | --- |
|  | Survey + open ended “why” comments | ***Activity 3 - POST ON TUESDAY, 8/10 - 9AM***  Have you ever had a serious conversation with a doctor, nurse, or other health professional about your wishes for your medical care if you become seriously ill, such as the types of treatments you do or don’t want to receive?  *Note: for this research, I’m defining serious illness as a disease or health issue such as cancer, heart disease or dementia that makes you feel sick enough that it’s increasingly hard to do your normal levels of work and activity.*  1. Yes  2. No [iTRACKS - IF PARTICIPANTS SAY NO - DIRECT THEM TO THIS FOLLOW UP QUESTION:  You indicated that you have not had a serious conversation with a doctor, nurse or other health professional about your wishes for your medical care if you became seriously ill. Would you want to? Tell me why or why not. |  |
|  | Survey + open ended “why” comments | ***Activity 4 – POST ON TUESDAY, 8/10 – 9AM [iTRACKS - PROGRAM SO THAT PARTICIPANTS COMPLETE ACTIVITY 3 BEFORE MOVING ON TO THIS ACTIVITY - ACTIVITY 4]***  Thinking more about serious illness (whether this is something you are currently living with or can imagine facing in the future) …  How worried would you be if you couldn’t speak for yourself that **your family/friends won't make the best/right decisions about your care?** Tell me *why* you feel this way in the space below.    Not at all worried  Not too worried  Somewhat worried  Very worried  **iTRACKS PROGRAMMING NOTES**: INCLUDE A FOLLOW-UP, OPEN-ENDED QUESTION FOR PARTICIPANTS TO ELABORATE ON WHY THEY INDICATED WHAT THEY DID |  |
| **Message testing** |  | ***At this point, participants will provide feedback on the calls to action message tests - specifically: community A will review/critique calls to action that are part of a public health marketing campaign; community B will review/critique calls to action included in a simple letter from a doctor’s office; community C will review/critique calls to action included in a more detailed letter coming from a doctor’s office (letter includes the “wrapper” language from June/July conversation and quality statement language).*** |  |
|  | **Message testing - PART 1:** highlighting exercises + open ended why comments | ***Activity 5 – POST ON TUESDAY, 8/10 – 3PM***  **COMMUNITY A**  **At this point in our research, we are going to focus our time on getting your thoughts and reactions to ideas and concepts … understanding what resonates with you, what’s believable and what would have the greatest impact on your health and wellbeing.**  To get into the right mindset for these next few activities, imagine seeing or hearing messaging that is part of a marketing campaign (such as a poster, a brochure or an ad on the radio) from a public health nonprofit organization that includes a recommended action that addresses your health and wellbeing. With this context in mind - let’s get started.   - **STEP 1:** There are four different actions in total - I’d like your feedback on each one. You’ll read each action - again, imagining that you are seeing this action in a marketing campaign (such as a poster, a brochure or an ad on the radio). - **STEP 2:**  Use a highlighting tool that includes 3 different colors (green = positive, yellow = neutral, red = negative) to indicate things you like, dislike and/or feel indifferent about within each action (could be the words and/or whole phrases). - **STEP 3:**  Add comments that explain *why* you indicated what you did. ***The comments are super important - please take the time to be as clear as you can.***   **iTRACKS PROGRAMMING NOTES:**   - LINK TO ACTUAL HIGHLIGHTING EXERCISE - RANDOMIZE ACTIONS FOR EACH PARTICIPANT TO ELIMINATE BIAS.   **COMMUNITY B**  **At this point in our research, we are going to focus our time on getting your thoughts and reactions to ideas and concepts … understanding what resonates with you, what’s believable and what would have the greatest impact on your health and wellbeing.**  To get into the right mindset for these next few activities, imagine that you’ve received a letter in the mail (or received an email) from your doctor’s office and in the letter (or email) there is a recommended action - something you may want to consider doing that in some way impacts your health and wellbeing. With this context in mind - let’s get started.   - **STEP 1:** There are three different letters in total - I’d like your feedback on each one. You’ll read each letter - again, imagining that you are receiving this letter or email from your doctor’s office. - **STEP 2:**  Use a highlighting tool that includes 3 different colors (green = positive, yellow = neutral, red = negative) to indicate things you like, dislike and/or feel indifferent about within each action (could be the words and/or whole phrases). - **STEP 3:**  Add comments that explain *why* you indicated what you did. ***The comments are super important - please take the time to be as clear as you can.***   **iTRACKS PROGRAMMING NOTES:**   - LINK TO ACTUAL HIGHLIGHTING EXERCISE - RANDOMIZE LETTERS FOR EACH PARTICIPANT TO ELIMINATE BIAS.   **COMMUNITY C**  **At this point in our research, we are going to focus our time on getting your thoughts and reactions to ideas and concepts … understanding what resonates with you, what’s believable and what would have the greatest impact on your health and wellbeing.**  To get into the right mindset for these next few activities, imagine that you’ve received a letter in the mail (or received an email) from your doctor’s office and in the letter (or email) there is a recommended action - something you may want to consider doing that in some way impacts your health and wellbeing. With this context in mind - let’s get started.   - **STEP 1:** There are two components to this imaginary letter - an opening paragraph and the recommended action. You’ll see three different versions of the letter - each includes a different recommended action. You’ll read each letter - again, imagining that you are receiving this from your doctor’s office. - **STEP 2:**  Use a highlighting tool that includes 3 different colors (green = positive, yellow = neutral, red = negative) to indicate things you like, dislike and/or feel indifferent about within each letter (could be the words and/or whole phrases). - **STEP 3:**  Add comments that explain *why* you indicated what you did. ***The comments are super important - please take the time to be as clear as you can.***   **iTRACKS PROGRAMMING NOTES:**   - LINK TO ACTUAL HIGHLIGHTING EXERCISE - RANDOMIZE LETTERS FOR EACH PARTICIPANT TO ELIMINATE BIAS.   **FOR COMMUNITY A - THE 4 ACTIONS TO BE USED FOR ACTIVITIES 5-8**  **You can speak up and have a say in your care**. Getting health care often involves choices that impact your life and wellbeing in different ways. Treatments only work if they work for you. For doctors to do their best job**,** they need to understand what is going on in your life, your priorities, and what matters to you. By asking your doctors to listen and by sharing with them what’s important, even if it’s hard, you can get better care. Ask your doctors to take time to help you weigh risks and rewards of treatments, and work with you to make sure your care is the best fit for you. The more you speak up, the better your care can be. There are many free resources that can help you think about what is important to you when it comes to health care, as well as ideas for questions to ask and things to share with your doctor.  **You can put your end of life affairs in order.** There are documents, instructions, and products such as life insurance, wills, and funeral directions, that will give the people around you the guidance and support they will need after you are gone. You can make it as easy and settled as you can for those you leave behind. You can ask friends and family members to refer you to trusted professionals or services, or find free resources online.  **You can make a plan for your health care in case you cannot speak for yourself**. Talk to a trusted person about becoming your health care proxy, a person who can make medical decisions for you if you can’t. Share with that person what matters most and what your good days look like. You may not be able to predict every choice that needs to be made, but you can give your trusted person the guiding principles to confidently make decisions for you if they have to. There are many free online resources that can help you pick a proxy and talk with this trusted person about how to choose the care that’s right for you.  **You can look for the right doctor.** A great doctor takes the time to treat you as a whole person, listens to what matters to you, is empathetic and non-judgmental, and gets to the root causes of your concerns to help improve your wellbeing. They know that the best care is collaborative with you and it happens when doctors and patients are working together, trusting each other, and committed to the patient’s wellbeing. If your needs and goals are not being met by your current doctors, you can look for other doctors. Some people who switch ask friends/family for referrals or look at doctors’ online reviews for recommendations.  **FOR COMMUNITY B - THE 3 SIMPLE LETTERS THAT INCLUDE RECOMMENDED ACTIONS TO BE USED FOR ACTIVITIES 5-8**  **Dear:**  As a valued member of our practice, we are writing to tell you about different things you can do to improve your care and wellbeing.  You can speak up and have a say in your care. Getting health care often involves choices that impact your life and wellbeing in different ways. Treatments only work if they work for you. For doctors to do their best job**,** they need to understand what is going on in your life, your priorities, and what matters to you. By asking your doctors to listen and by sharing with them what’s important, even if it’s hard, you can get better care. Ask your doctors to take time to help you weigh risks and rewards of treatments, and work with you to make sure your care is the best fit for you. The more you speak up, the better your care can be. There are many free resources that can help you think about what is important to you when it comes to health care, as well as ideas for questions to ask and things to share with your doctor.  **Dear:**  As a valued member of our practice, we are writing to tell you about different things you can do to improve your care and wellbeing.  You can put your end of life affairs in order. There are documents, instructions, and products such as life insurance, wills, and funeral directions, that will give the people around you the guidance and support they will need after you are gone. You can make it as easy and settled as you can for those you leave behind. You can ask friends and family members to refer you to trusted professionals or services, or find free resources online.  **Dear**:  As a valued member of our practice, we are writing to tell you about different things you can do to improve your care and wellbeing.  You can make a plan for your health care in case you cannot speak for yourself**.** Talk to a trusted person about becoming your health care proxy, a person who can make medical decisions for you if you can’t. Share with that person what matters most and what your good days look like. You may not be able to predict every choice that needs to be made, but you can give your trusted person the guiding principles to confidently make decisions for you if they have to. There are many free online resources that can help you pick a proxy and talk with this trusted person about how to choose the care that’s right for you.  **FOR COMMUNITY C - THE 3 DETAILED LETTERS (WITH FRAMING INTRO) THAT INCLUDE RECOMMENDED ACTIONS TO BE USED FOR ACTIVITIES 5-8**  **Dear:**  At our practice, we know that the best care is collaborative with you. It requires learning about you, and what is going on in your life, so we can address not just the symptoms but the root cause of why you came in. We also recognize that a lot of the things that impact your wellbeing are not directly related to health or health care. Which is why we take the time to listen and make sure whatever we decide on together, really works for you. In the meantime, there are non-health related things you can do that could have a positive impact on your life.  You can put your end of life affairs in order**.** There are documents, instructions, and products such as life insurance, wills, and funeral directions, that will give the people around you the guidance and support they will need after you are gone. You can make it as easy and settled as you can for those you leave behind. You can ask friends and family members to refer you to trusted professionals or services, or find free resources online.  **Dear:**  As a member of our practice, we are writing to check in. We know this is a complicated and challenging time for many people. But there are a lot of resources and programs that can help improve your wellbeing, both within our practice and in your community. From finding a person to talk with about your mental health, to meal or transportation support, or help with jobs and housing, there are many places to find support. We have staff that are knowledgeable about these things and can help connect you to helpful resources.  There are also things you can do to improve your care and wellbeing. You can make a plan for your health care in case you cannot speak for yourself. Talk to a trusted person about becoming your health care proxy, a person who can make medical decisions for you if you can’t. Share with that person what matters most and what your good days look like. You may not be able to predict every choice that needs to be made, but you can give your trusted person the guiding principles to confidently make decisions for you if they have to. There are many free online resources that can help you pick a proxy and talk with this trusted person about how to choose the care that’s right for you.  **Dear:**  At our practice, we’re committed to care that is kind, empathetic, patient, and non-judgmental. Care that treats you like a whole person. We know that everyone is important, worthy of care, of being listened to and believed, and being treated with dignity and respect. Your voice, your questions, and your concerns are always welcome.  There are things you can do so that, together, we can figure out what is best for you, no matter the situation. You can speak up and have a say in your care. Getting health care often involves choices that impact your life and wellbeing in different ways. Treatments only work if they work for you. For doctors to do their best job**,** they need to understand what is going on in your life, your priorities, and what matters to you. By asking your doctors to listen and by sharing with them what’s important, even if it’s hard, you can get better care. Ask your doctors to take time to help you weigh risks and rewards of treatments, and work with you to make sure your care is the best fit for you. The more you speak up, the better your care can be. There are many free resources that can help you think about what is important to you when it comes to health care, as well as ideas for questions to ask and things to share with your doctor. |  |
|  | **Message testing- PART 2:**  surveys + open ended “why”  comments | ***Activity 6 – POST ON WEDNESDAY, 8/11 – 9AM***  **COMMUNITY A**  Let’s stick with the context I introduced in the last activity … imagine that you are seeing or hearing messaging that is part of a marketing campaign (such as a poster, a brochure or an ad on the radio) from a public health non profit organization that includes a recommended action that addresses your health and wellbeing..  I’m going to share each of the four actions again and ask that you share your thoughts on **believability** (how much you truly buy into what’s being said), **difficulty** (the degree to which you can actually do what’s being said) and **impact** (looking into the future - the degree to which what’s being said is going to positively impact your health and wellbeing).  We’ll start with **believability** … [show each action separately - include rating language and follow up with open ended why]  Using the following scale where 1 = not at all believable, 2 = somewhat unbelievable, 3 = somewhat believable, and 4 = very believable - how believable is the recommended action? Tell me why you indicated what you did.  **FOLLOW UP WITH:**  Looking at all four actions - which of the four is the **most believable**?  [include all 4 actions - allow participant to only choose one]  **iTRACKS PROGRAMMING NOTES:** RANDOMIZE ACTIONS FOR EACH PARTICIPANT TO ELIMINATE BIAS.  **COMMUNITY B**  Let’s stick with the context I introduced in the last activity … imagine that you’ve received a letter in the mail (or received an email) from your doctor’s office and in the letter (or email) there is a recommended action - something you may want to consider doing that in some way impacts your health and wellbeing.  I’m going to share each of the three letters again and ask that you share your thoughts on **believability** (how much you truly buy into what’s being said), **difficulty** (the degree to which you can actually do what’s being said) and **impact** (looking into the future - the degree to which what’s being said is going to positively impact your health and wellbeing).  We’ll start with **believability** … [show each letter separately - include rating language and follow up with open ended why]  Using the following scale where 1 = not at all believable, 2 = somewhat unbelievable, 3 = somewhat believable, and 4 = very believable - how believable is the recommended action? Tell me why you indicated what you did.  **FOLLOW UP WITH:**  Looking at all of the letters - which of the three is the **most believable**?  [include all 3 letters - allow participant to only choose one]  **iTRACKS PROGRAMMING NOTES:** RANDOMIZE LETTERS FOR EACH PARTICIPANT TO ELIMINATE BIAS.    **COMMUNITY C**  Let’s stick with the context I introduced in the last activity … imagine that you’ve received a letter in the mail (or received an email) from your doctor’s office and in the letter (or email) there is a recommended action - something you may want to consider doing that in some way impacts your health and wellbeing.  I’m going to share each of the three letters again and ask that you share your thoughts on **believability** (how much you truly buy into what’s being said), **difficulty** (the degree to which you can actually do what’s being said) and **impact** (looking into the future - the degree to which what’s being said is going to positively impact your health and wellbeing).  We’ll start with **believability** … [show each letter separately - include rating language and follow up with open ended why]  Using the following scale where 1 = not at all believable, 2 = somewhat unbelievable, 3 = somewhat believable, and 4 = very believable - how believable is the recommended action included in the letter? Tell me why you indicated what you did.  **FOLLOW UP WITH:**  Looking at all of the letters - which of the three is the **most believable**?  [include all 3 letters - allow participant to only choose one]  **iTRACKS PROGRAMMING NOTES:** RANDOMIZE LETTERS FOR EACH PARTICIPANT TO ELIMINATE BIAS. |  |
|  |  | ***Activity 7 - POST ON WEDNESDAY, 8/11 – 3PM***  **COMMUNITY A**  Now let’s focus on **difficulty** … [show each action separately - include rating language and follow up with open ended why]  Using the following scale where 1 = very difficult, 2 = somewhat difficult, 3 = somewhat easy, and 4 = very easy - how easy or difficult is it for you to do the recommended action? Tell me why you indicated what you did.  **FOLLOW UP WITH:**  Looking at all four actions - which of the four is the ***most*** difficult?  [include all 4 actions - allow participant to only choose one]  **iTRACKS PROGRAMMING NOTES:** RANDOMIZE ACTIONS FOR EACH PARTICIPANT TO ELIMINATE BIAS.  **COMMUNITY B**  Now let’s focus on **difficulty** … [show each letter separately - include rating language and follow up with open ended why]  **FOLLOW UP WITH:**  Looking at all three letters that include recommended actions - which of the three recommended actions is ***most*** difficult?  **iTRACKS PROGRAMMING NOTES:** RANDOMIZE LETTERS FOR EACH PARTICIPANT TO ELIMINATE BIAS.  **COMMUNITY C**  Now let’s focus on **difficulty** … [show each letter separately - include rating language and follow up with open ended why]  **FOLLOW UP WITH:**  Looking at all three letters that include recommended actions - which of the three recommended actions is the ***most*** difficult?  **iTRACKS PROGRAMMING NOTES:** RANDOMIZE LETTERS FOR EACH PARTICIPANT TO ELIMINATE BIAS. |  |

|  |  | ***Activity 8 – POST ON THURSDAY, 8/12 – 9AM***  **COMMUNITY A**  Now let’s focus on **impact** … [show each action separately - include rating language and follow up with open ended why]  **FOLLOW UP WITH:**  Looking at all four actions - which of the four do you believe would have the ***greatest positive impact*** on your health and wellbeing?  [include all 4 actions - allow participant to only choose one]  **iTRACKS PROGRAMMING NOTES:** RANDOMIZE ACTIONS FOR EACH PARTICIPANT TO ELIMINATE BIAS.  **COMMUNITY B**  Now let’s focus on **impact** … [show each letter separately - include rating language and follow up with open ended why]  **FOLLOW UP WITH:**  Looking at all three recommended actions included in the letters - which of the three do you believe would have the ***greatest positive impact*** on your health and wellbeing?  [include all 3 letters - allow participant to only choose one]  **iTRACKS PROGRAMMING NOTES:** RANDOMIZE LETTERS FOR EACH PARTICIPANT TO ELIMINATE BIAS.  **COMMUNITY C**  Now let’s focus on **impact** … [show each letter separately - include rating language and follow up with open ended why]  Using the following scale where 1 = no impact at all, 2 = very little impact, 3 = some impact, and 4 = big impact - how much of a positive impact do you think the recommended action included in the letter would have on your health and wellbeing? Tell me why you indicated what you did.  **FOLLOW UP WITH:**  Looking at all three letters that include the recommended actions - which of the three recommended actions do you believe would have the ***greatest positive impact*** on your health and wellbeing?  [include all 3 letters - allow participant to only choose one]  **iTRACKS PROGRAMMING NOTES:** RANDOMIZE LETTERS FOR EACH PARTICIPANT TO ELIMINATE BIAS. |  |
| --- | --- | --- | --- |

| **Wrap up** |  |  |  |
| --- | --- | --- | --- |
|  | Discussion board | ***Final activity – POST ON THURSDAY, 8/12 – 3PM***  Well – it’s hard to believe **we’re at the end of our week already!** The ground we’ve covered is amazing – and your stories, opinions and ideas are impressive and clearly come from your heart.  Let’s end with two key questions …  Reflect back on the various messages and actions you’ve reviewed over the last few days:   1. **What one message or action has inspired you to do something once this community ends?** *Why* this message or action vs. others? 2. **What one message or action do you think the Massachusetts Coalition for Serious Illness Care should prioritize *most*** given their mission to create positive health care experiences, especially for people with serious illnesses when it matters most? *Why* this message or action vs. others?   **On behalf of the Massachusetts Coalition for Serious Illness Care, thank you again for the time and effort you’ve put into this exploration.** |  |
